# Third-generation cephalosporin use is frequently non-guideline-concordant in severe community-acquired pneumonia: Findings from a French critical care cohort

**DOI:** 10.1371/journal.pone.0354339

**Published:** 2026-07-29

**Authors:** Quitterie Lostie de Kerhor, Zeina Sabouni, Cyrille Geay, Catherine Bouvet-Velly, Laurent Ducros, Jonathan Chelly

**Affiliations:** 1 Emergency Department, Centre Hospitalier Intercommunal Toulon La Seyne Sur Mer, Toulon, France; 2 Bacteriological Laboratory, Centre Hospitalier Intercommunal Toulon La Seyne Sur Mer, Toulon, France; 3 Intensive Care Unit, Centre Hospitalier Intercommunal Toulon La Seyne Sur Mer, Toulon, France; 4 Clinical Research Unit, Délégation à la Recherche Clinique et à l’Innovation (DRCI) du Groupement Hospitalier Territorial (GHT) du Var, Centre Hospitalier Intercommunal Toulon La Seyne Sur Mer, Toulon, France; 5 Centre d’étude et de Recherche sur les services de santé et la qualité de vie (CEReSS), Aix-Marseille Université, Marseille, France; Fayetteville State University, UNITED STATES OF AMERICA

## Abstract

**Background and objectives:**

Severe community-acquired pneumonia (CAP) remains a major cause of morbidity and mortality, requiring prompt empirical antibiotic therapy. The choice between amoxicillin/clavulanic acid (AMC) and third-generation cephalosporin (3GC) as first-line beta-lactam therapy in the ICU is still debated. To assess AMC and 3GC susceptibility in severe CAP or community-acquired aspiration pneumonia (CAAP) caused by *Streptococcus pneumoniae (SP)*, *Haemophilus influenzae* (HI) and/or *Staphylococcus aureus (SA)*, and to evaluate the appropriateness of empirical antibiotic therapy.

**Methodology:**

We conducted a single-center retrospective study including patients admitted to the ICU between 01/01/2018 and 30/11/2022 for severe CAP/CAAP with microbiological documentation of at least one of the targeted pathogens. Clinical, microbiological, therapeutic, and outcome data were collected. The primary endpoint was AMC and 3GC susceptibility rates (expressed as percentage and [95% confidence intervals]).

**Principal findings:**

Among 104 included patients (median age 64 [48–71] years; 67% male), 60 (57.7%) had CAAP. AMC susceptibility rates were 81.5% [61.9–93.7], 78.0% [62.4–89.4], and 100% [92.6–100.0], while 3GC susceptibility rates were 96.3% [81.0–99.9], 95.1% [83.5–99.4], and 100% [92.6–100.0], for *SP*, *HI*, and *SA*, respectively. AMC was the empirical treatment in 59% of cases, and this choice was microbiologically appropriate in 86.9% of these prescriptions. Conversely, 3GC were prescribed empirically in 20% of cases, but this broad-spectrum choice was unjustified in 80% of those situations. Antibiotic de-escalation was performed in 28% of cases.

**Conclusion:**

In this single-center retrospective cohort, AMC appears to be a relevant empirical option for severe CAP/CAAP in the ICU, while 3GCs seem frequently overused. These findings required prospective multicenter validation before practice change can be recommended.

## Introduction

With more than 489 million lower respiratory tract infections worldwide in 2019 and an annual global incidence ranging from 1 to 25 cases per 1000 inhabitants [1–3], community-acquired pneumonia (CAP) remains a major cause of morbidity and mortality. Its management, particularly optimization of empirical antibiotic therapy, represents a daily challenge for emergency and critical care physicians. Approximately 40% of patients with CAP require hospitalization, and around 5% require intensive care unit (ICU) admission [3]. CAP is defined as severe when ICU admission is required, most frequently due to acute respiratory failure requiring mechanical ventilation (MV) and/or the presence of septic shock requiring vasopressors. It is crucial to distinguish severe CAP from non-severe forms, as it warrants separate therapeutic consideration. Indeed, the high immediate mortality risk associated with severe CAP (reaching up to 30% in patients under MV) [1,4] dictates the need for prompt and often broader empirical antibiotic therapy upon admission, differing substantially from standard medical ward protocols. Community-acquired aspiration pneumonia (CAAP) represents a specific subgroup of CAP, accounting for 5.1 to 38.2% of cases and is related to the aspiration of oropharyngeal and/or gastric contents into the lower respiratory tract. Epidemiological data remain limited due to the absence of robust diagnostic criteria, and diagnosis is usually presumptive, based on clinical and anamnestic elements [5]. CAAP may result from micro-aspiration (e.g., during sleep, particularly in elderly patients, or those with prior stroke) or macro-aspiration, which is more often symptomatic and associated with impaired consciousness, swallowing disorders, gastroesophageal reflux, or defective protective airway reflexes. CAAP is frequently evaluated alongside severe CAP in the ICU setting. This grouping is justified because both conditions present with overlapping acute respiratory failure, are often difficult to reliably distinguish upon initial admission, and are managed under similar initial severity protocols. The main pyogenic bacteria implicated in severe CAP and CAAP are *Streptococcus pneumoniæ*, *Staphylococcus aureus*, and *Hæmophilus influenzæ*, in addition to *Pseudomonas æruginosa* in selected patients with risk factors (e.g., severe chronic respiratory disease) [1,5–7].

Beyond respiratory and hemodynamic management, prompt initiation of appropriate empirical antibiotic therapy remains crucial in the treatment of severe CAP or CAAP. French and international guidelines emphasize early empirical therapy tailored to local bacterial ecology and resistance patterns [5,8]. In addition to the systematic association of a macrolide to cover atypical pathogens, the choice of the beta-lactam remains controversial. French guidelines recommend third-generation cephalosporins (3GCs) as first-line therapy in severe CAP [8], whereas American guidelines allow either a 3GC or a high-dose penicillin–beta-lactamase inhibitor combination [9]. For CAAP, recent French guidelines recommend amoxicillin-clavulanate (AMC) even in severe cases, provided there is no risk factor for *P. aeruginosa* [5].

The use of 3GCs has been justified by the presence of *S. pneumoniæ* strains with reduced susceptibility to penicillin, though their incidence is declining and mostly related to non-vaccine serotypes. Regarding *H. influenzæ*, 20–25% of isolates are estimated to be resistant to AMC, although this rate has recently decreased [10]; these resistant strains, however, have also been reported as mostly resistant to 3GCs (69%) [11].

Although AMC has demonstrated favorable outcomes in non-severe CAP [12], its role in severe forms remains poorly investigated. AMC may represent a relevant alternative to 3GCs, offering comparable coverage of methicillin-susceptible *S. aureus*, improved anaerobic coverage, activity against most penicillin-resistant *S. pneumoniae* at high doses, potential ecological benefits [6,13], and the possibility of an oral switch after clinical improvement.

To date, no robust study has compared these two empirical regimens in severe CAP or CAAP. Previous studies reported high rates of unjustified empirical 3GCs use in hospitalized and ICU patients [6,14].

We therefore conducted a retrospective observational study including patients admitted to the ICU for severe CAP or CAAP documented with *S. pneumoniæ*, *H. influenzæ*, and/or *S. aureus*. Our objectives were to describe AMC and 3GC susceptibility profiles, evaluate empirical antibiotic use and subsequent adaptation, and identify potential risk factors for AMC resistance.

## Materials and methods

### Study setting and population

We conducted a retrospective, single-center study including consecutive adult patients admitted between 01/01/2018 and 30/11/2022 to the mixed ICU of a French non-academic tertiary referral hospital (Centre Hospitalier Intercommunal Toulon La Seyne-sur-Mer, Sainte Musse Hospital). The department comprises 16 ICU beds and 8 intermediate care beds. Patients were included if they were admitted for severe CAP or CAAP diagnosed within 48 hours of hospital admission. The study period was specifically chosen to evaluate contemporary bacterial ecology and baseline empirical antibiotic prescribing habits prior to the anticipated implementation of updated national guidelines and subsequent local antimicrobial stewardship protocols. Inclusion required microbiological documentation of *S. pneumoniæ*, *H. influenzæ*, and/or *S. aureus* with available antimicrobial susceptibility testing. Severity was defined according to French CAP guidelines [8]. Patients hospitalized within the three months preceding admission, patients with another documented infection within 48 hours after admission, patients with late microbiological sampling (>48 hours), and patients who died or were discharged before bacteriological documentation were excluded. Ventilator-associated pneumonia and healthcare-associated pneumonia (including nursing home residents or patients receiving home intravenous therapy) were also excluded. Immunocompromised patients (such as transplant recipients, patients with HIV, or those undergoing active chemotherapy) were excluded by definition, as they do not meet the diagnostic criteria for CAP or CAAP, and were therefore not evaluated during initial screening. Regarding polymicrobial infections, only cases involving the targeted pathogens were included. Finally, patients admitted for organ donation, those under legal protection measures, or those with incomplete microbiological or clinical records were excluded.

The study was supported by the *Centre Hospitalier Intercommunal de Toulon La Seyne-sur-Mer*, approved by the local Ethics Committee on 06/03/2025 (Institutional Review Board number 00012962), and registered retrospectively at *ClinicalTrials.gov* on 10/02/2026 (NCT07322549). Patient information and data use complied with French legislation and the Declaration of Helsinki (1975). This study followed the STROBE guidelines for reporting observational studies [15].

### Data collection and outcome variables

Data were retrospectively extracted, between 07/03/2025 and 30/09/2025, from the hospital’s electronic medical records, and were strictly pseudonymized prior to analysis. Each patient was assigned a unique study identification number, and the correspondence key linking patient identities to these numbers was stored separately on a secure, restricted-access server hosted by the hospital. Consequently, all subsequent data handling and statistical analyses were performed on a fully de-identified dataset to ensure patient confidentiality. Collected variables included patients’ baseline characteristics, comorbidities, Pneumonia Severity Index (PSI), and Simplified Acute Physiology Score II (SAPS II). Microbiological data included the type of sample, identified pathogens, susceptibility to AMC and 3GCs, and the presence of respiratory viral co-infection. Antimicrobial susceptibility testing was performed by the microbiological laboratory using routine disk diffusion methods, and results were interpreted according to the European Committee on Antimicrobial Susceptibility Testing (EUCAST) breakpoints and the recommendations of the Antibiogram Committee of the French Society of Microbiology (CA-SFM) [16]. All susceptibility profiles were systematically validated by a medical bacteriologist. The clinical relevance of the empirical beta-lactam therapy was evaluated retrospectively by the investigators, based on consensus agreement between a medical bacteriologist and an ICU physician. All empirical antibiotics therapies and secondary adaptations used to treat the documented CAP or CAAP were recorded. Based on microbiological results, secondary antibiotic adaptation was classified as continuation, de-escalation, or escalation. Continuation was defined as maintenance of the initial empirical antibiotic regimen without any change in the beta-lactam spectrum. De-escalation was defined as a switch to an antibiotic with a narrower spectrum of activity. Escalation was defined as a switch to a broader spectrum antibiotic regimen due to resistance or inadequate coverage. Classification focused on the beta-lactam component of therapy. Dose adjustments were not considered as escalation or de-escalation, as all patients treated with high-dose regimens consistent with ICU management of severe pneumonia. Clinical outcomes included the need for mechanical ventilation, occurrence of shock, nosocomial infections, colonization with multidrug-resistant bacteria, and ICU mortality. The primary outcome was the susceptibility rates of documented pathogens to AMC and 3GCs. Secondary outcomes included comparison of AMC-susceptible (AMC-S) versus AMC-resistant (AMC-R) cases, and appropriateness of empirical antibiotic prescriptions (appropriate, inappropriate, unjustified). Empirical beta-lactam therapy was classified as appropriate, inappropriate, or unjustified based on susceptibility and beta-lactam spectrum hierarchy (AMC < 3GCs < piperacillin-tazobactam (PTZ)). Prescribed empirical therapy was classified as appropriate if the antibiotic effectively covered the isolated pathogen based on susceptibility testing, and inappropriate if the pathogen was resistant to the treatment. Among appropriate prescriptions, the use of a broader-spectrum agent (3GCs or PTZ) was classified as justified if the pathogen exhibited resistance to narrower-spectrum alternatives. Conversely, it was classified as unjustified when the empirical antibiotic was active, but a narrower-spectrum recommended option would have provided identical coverage, representing a situation of overtreatment.

### Statistical analysis

Continuous variables are expressed as median [interquartile range], while categorical variables are expressed as absolute numbers and percentages. The assumption of normality for continuous data was evaluated via a visual inspection of histograms. Because the distributions were found to be non-normal, continuous variables were compared between groups using the non-parametric Mann-Whitney U test. Categorical variables were compared using Fisher’s exact test. Regarding the primary outcome, confidence intervals (CIs) for proportions were calculated at the 95% confidence level using the exact binomial method (Clopper-Pearson). A p-value <0.05 was considered statistically significant. Statistical analyses were performed using R++ software (version 1.8.04, Toulouse, France).

## Results

### Study population

**Fig 1:**
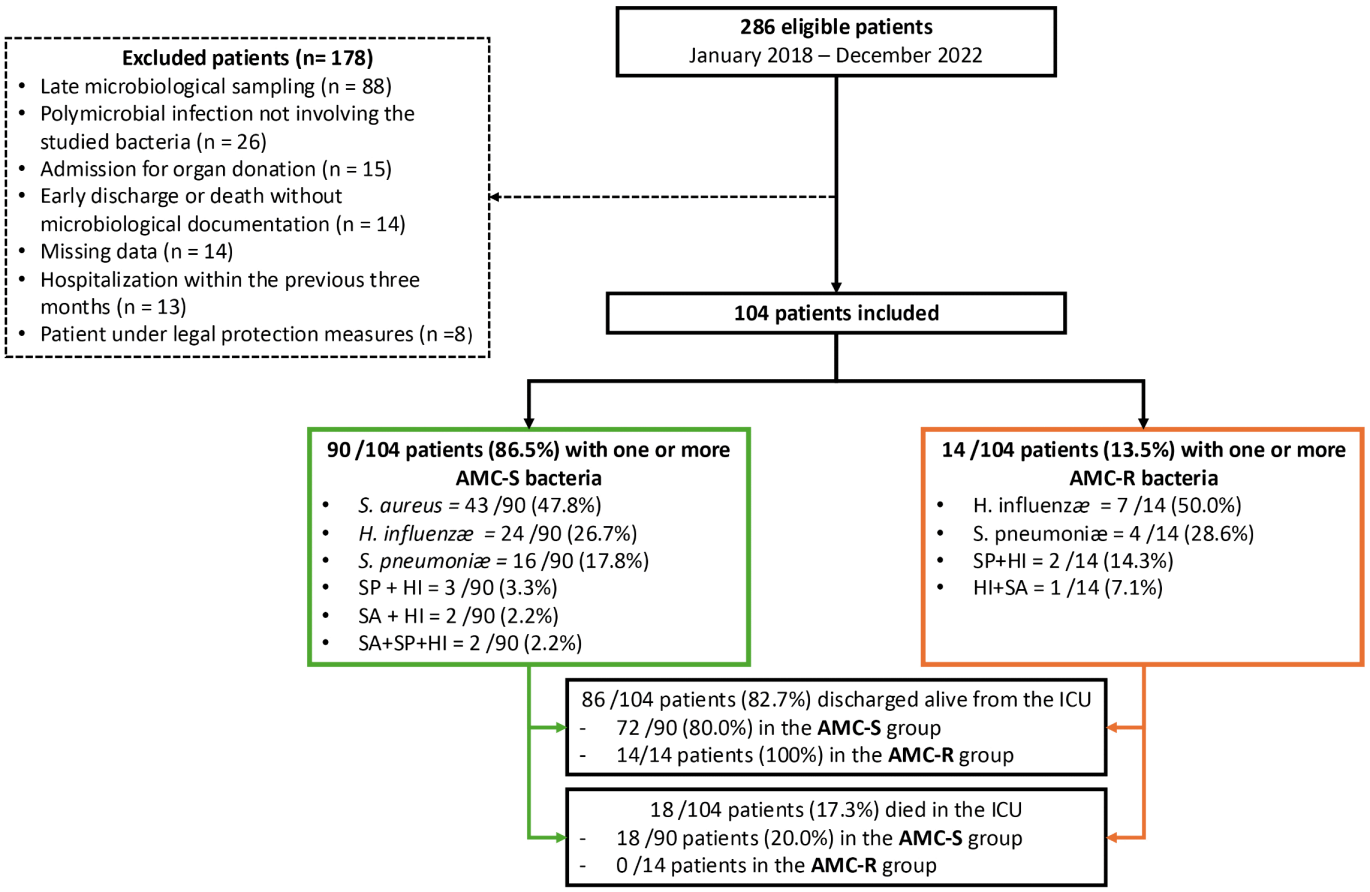
Study flow chart and classification of included patients according to amoxicillin-clavulanate susceptibility. AMC-S: susceptibility to amoxicillin-clavulanate; AMC-R: resistance to amoxicillin-clavulanate; SA: *Staphylococcus aureus*; HI: *Hæmophilus influenzæ*; SP: *Streptococcus pneumoniæ*; ICU: intensive care unit.

During the study period, 286 patients were admitted to the ICU with CAP or CAAP, of whom 104 were included in the final cohort (Fig 1). Baseline characteristics are summarized in Table 1. Median age was 64 [48–71] years, and 67% were male. CAAP accounted for 57.7% of cases. MV was required in 73.1% of patients, and shock occurred in 19.2%. Median SAPS II and PSI were 48 [35–59] and 142 [119–171], respectively.

**Table 1:** Baseline characteristics of the overall cohort.

Characteristics	Overall cohort N = 104
**Age** – years	64 [48–71]
**Sex –** Male/Female	70 (67.3)/34 (32.7)
**Body mass index** – kg/m^2^	25 [22–29]
**Chronic respiratory disease**	41 (39.4)
**Smoking history**	66 (65.4)
**Chronic alcohol abuse**	24 (23.1)
**Charlson comorbidity index**	3 [1–5]
**SAPS II**	48 [35–59]
**PSI**	142 [119–171]
**PSI risk stratification**	
Low risk (<91)	9 (8.7)
Moderate risk (91–130)	27 (26.0)
High risk (PSI > 130)	68 (65.4)
**Antibiotic therapy before hospital admission**	3 (2.9)
**Antibiotic therapy within the previous 3 months**	4 (4.0)
**Reason for ICU admission**	
Respiratory	45 (43.3)
Neurological	30 (28.9)
Toxicological	16 (15.4)
Metabolic	4 (3.9)
Gastrointestinal	3 (2.9)
Cardiovascular	2 (1.9)
Trauma	1 (1.0)
Other	3 (2.9)
**Community-acquired aspiration pneumonia**	60 (57.7)

Data are expressed as median [interquartile range, 25th-75^th^ percentile] for continuous variables and as number (percentage) for categorical variables. SAPS II: Simplified Acute Physiology Score II; PSI: Pneumonia Severity Index (Fine score); ICU: intensive care unit.

### Microbiological findings and susceptibility profiles

**Fig 2:**
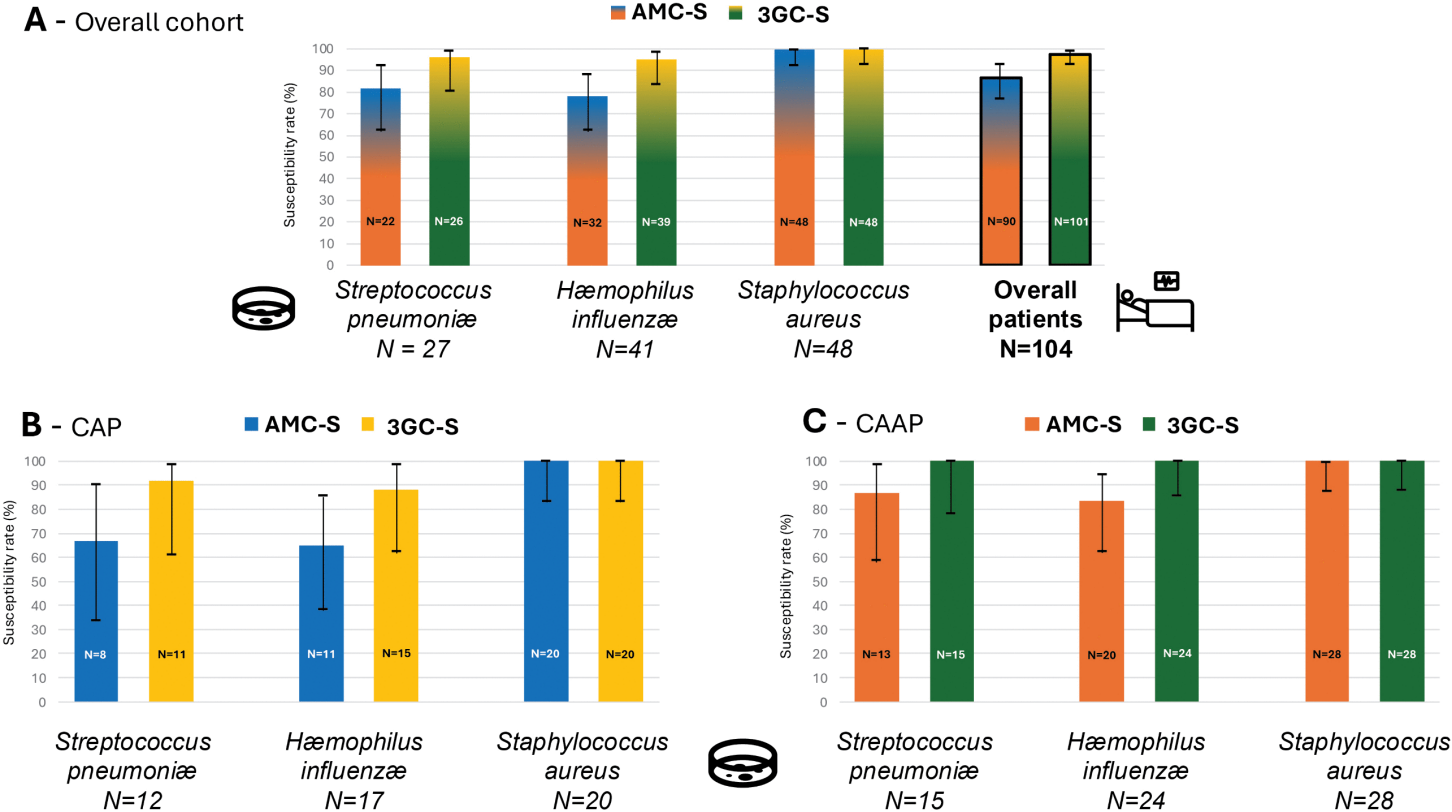
Antimicrobial susceptibility profiles to amoxicillin-clavulanate and third-generation cephalosporins. Panel A: Displays susceptibility profiles in the overall cohort, by pathogen for the three categories on the left (N = 116), and by patient (N = 104) for the category on the right (bold borders). Panels B and C: Display pathogen-specific susceptibility profiles among CAP and CAAP, respectively. Error bars represent 95% confidence intervals; sample sizes (N) are detailed below each category. CAP: community-acquired pneumonia; CAAP: community-acquired aspiration pneumonia; AMC-S: amoxicillin-clavulanate susceptible; 3GC-S: third-generation cephalosporin susceptible.

Microbiological identification was obtained mainly from respiratory samples (95.7%), more than two-thirds of which were tracheal aspirates. Overall, 86.5% [78.4–92.4] of patients were infected with pathogens fully susceptible to AMC, whereas 97.1% [91.6–99.4] were fully susceptible to 3GCs (Fig 1). Susceptibility profiles according to the identified pathogen are shown in Fig 2. AMC susceptibility rates for *S. pneumoniæ*, *H. influenzæ* and *S. aureus* were 81.5% [61.9–93.7], 78.0% [62.4–89.4], and 100% [92.6–100.0], while 3GC susceptibility rates were 96.3% [81.0–99.9], 95.1% [83.5–99.4], and 100% [92.6–100.0], respectively.

### Comparison between AMC-S and AMC-R groups

No significant differences were observed between both groups regarding baseline characteristics. There was a non-significant trend toward higher PSI score in the AMC-S group compared with the AMC-R group (145 [120–177] vs. 133 [107–137], respectively; p = 0.06), as well as a higher proportion of CAAP (61.1% vs. 35.7%, respectively; p = 0.08) (S1 Table). *S. aureus* was more frequently identified in the AMC-S group (52.2% vs. 0%; p = 0.001). Conversely, *H. influenzæ* was more frequently identified in the AMC-R group (71.4% vs. 34.4%; p = 0.02) (Table 2). Eighteen patients (17.3%) had a documented viral co-infection, mainly related to SARS-CoV-2, with no significant difference between AMC-S and AMC-R groups.

**Table 2:** Comparison of patients’ characteristics and microbiological findings according to amoxicillin-clavulanate susceptibility.

Parameters	AMC-R (N = 14)	AMC-S (N = 90)	p value
**Bacterial identification**			
*S. pneumoniæ*	6 (42.9)	21 (23.3)	0.18
*H. influenzæ*	10 (71.4)	31 (34.4)	0.02
*S. aureus*	0 (0)	47 (52.2)	0.001
**Viral co-infection**	3 (21.4)	15 (16.7)	0.70
SARS-CoV-2	3 (21.4)	11 (12.2)	0.39
Influenza	0 (0)	3 (3.3)	1.00
Other	0 (0)	1 (1.1)	1.00

Variables are expressed as number (percentage) and compared using Fischer’s exact test. AMC-R: amoxicillin-clavulanate resistant; BAL: bronchoalveolar lavage.

### Empirical antibiotic therapy and microbiological documentation

Overall, 78 patients (75.0%) received empirical monotherapy. The most frequently used empirical beta-lactams were AMC, 3GCs, and PTZ, used in 58.7%, 19.2%, and 16.3% of cases, respectively. AMC was the most frequently prescribed agent in monotherapy (75.6%), whereas 3GCs were more often used as part of combination regimens. Diagnostic and therapeutic management according to the identified pathogen are detailed in Table 3. Empirical antibiotic therapy, regardless of the molecule or combination, was considered appropriate in 92 patients (88.5%). Three patients (2.9%) did not receive any empirical antibiotic therapy.

**Table 3:** Microbiological documentation and antibiotic management according to the identified pathogen (116 bacterial documentations among the 104 included patients*).

Parameters	SA N = 48	HI N = 41	SP N = 27
**Type of microbiological documentation**			
Respiratory sample	47 (97.1)	39 (95.1)	25 (92.6)
*Tracheal aspirate*	31 (64.6)	31 (75.6)	18 (66.7)
*Sputum culture*	14 (29;1)	7 (17.1)	7 (25.9)
*Bronchoalveolar lavage*	2 (4.2)	1 (2.4)	0 (0)
Blood culture	1 (2.1)	2 (4.9)	2 (7.4)
**Time from hospital admission to microbiological sampling –** days	1 [0–2]	1 [0–2]	1 [0–1]
**Empiric antibiotic therapy**			
Monotherapy	37 (77.1)	30 (73.2)	21 (77.8)
Dual therapy	9 (18.8)	8 (19.5)	4 (14.8)
Triple therapy	0 (0)	2 (4.9)	1 (3.7)
None	2 (4.2)	1 (2.4)	1 (3.7)
**Empirical anti-pyogenic antibiotic therapy**			
AMC	28 (58.3)	24 (58.5)	17 (63.0)
C3G	7 (14.5)	8 (19.5)	5(18.5)
PTZ	9 (18.8)	8 (19.5)	3 (11.1)
Amoxicillin	0 (0)	0 (0)	1 (3.7)
Linezolid	1 (2.1)	0 (0)	0 (0)
Oxacillin	1 (2.1)	0 (0)	0 (0)
**Associated empirical antibiotic in dual or triple therapy**			
Levofloxacin	1 (2.1)	3 (7.3)	4 (14.8)
Metronidazole	4 (8.3)	3 (7.3)	0 (0)
Aminoglycoside	2 (4.2)	2 (4.9)	1 (3.7)
Spiramycin	2 (4.2)	2 (4.9)	0 (0)
Linezolid	0 (0)	2 (4.9)	1 (3.7)
**Antimicrobial susceptibility profile**			
AMC susceptible	48 (100.0)	32 (78.0)	22 (81.5)
3GC susceptible	48 (100.0)	39 (95.1)	26 (96.3)
Empirical therapy adapted to susceptibility results **	46 (95.8)	34 (82.9)	24 (85.2)
**Secondary antibiotic adaptation**			
No modification	27 (56.3)	21 (51.2)	16 (59.3)
De-escalation	18 (37.5)	15 (36.6)	8 (29.6)
Escalation	3 (6.2)	5 (12.2)	3 (11.1)

Continuous variables are expressed as median [interquartile range, 25^th^–75^th^ percentile], and categorical variables as number (percentage). SP: *Streptococcus pneumoniæ*; HI: *Hæmophilus influenzæ*; SA: *Staphylococcus aureus*; AMC: amoxicillin-clavulanate; 3GC: third-generation cephalosporin; PTZ: piperacillin-tazobactam.

* Ten patients had polymicrobial documentation involving studied pathogens: five with SP + HI, three with HI + SA, and two with SP + HI + SA.

** Three patients did not receive any empirical antibiotic therapy (two with SA, and one with SP).

### Secondary antibiotic adaptation

**Fig 3:**
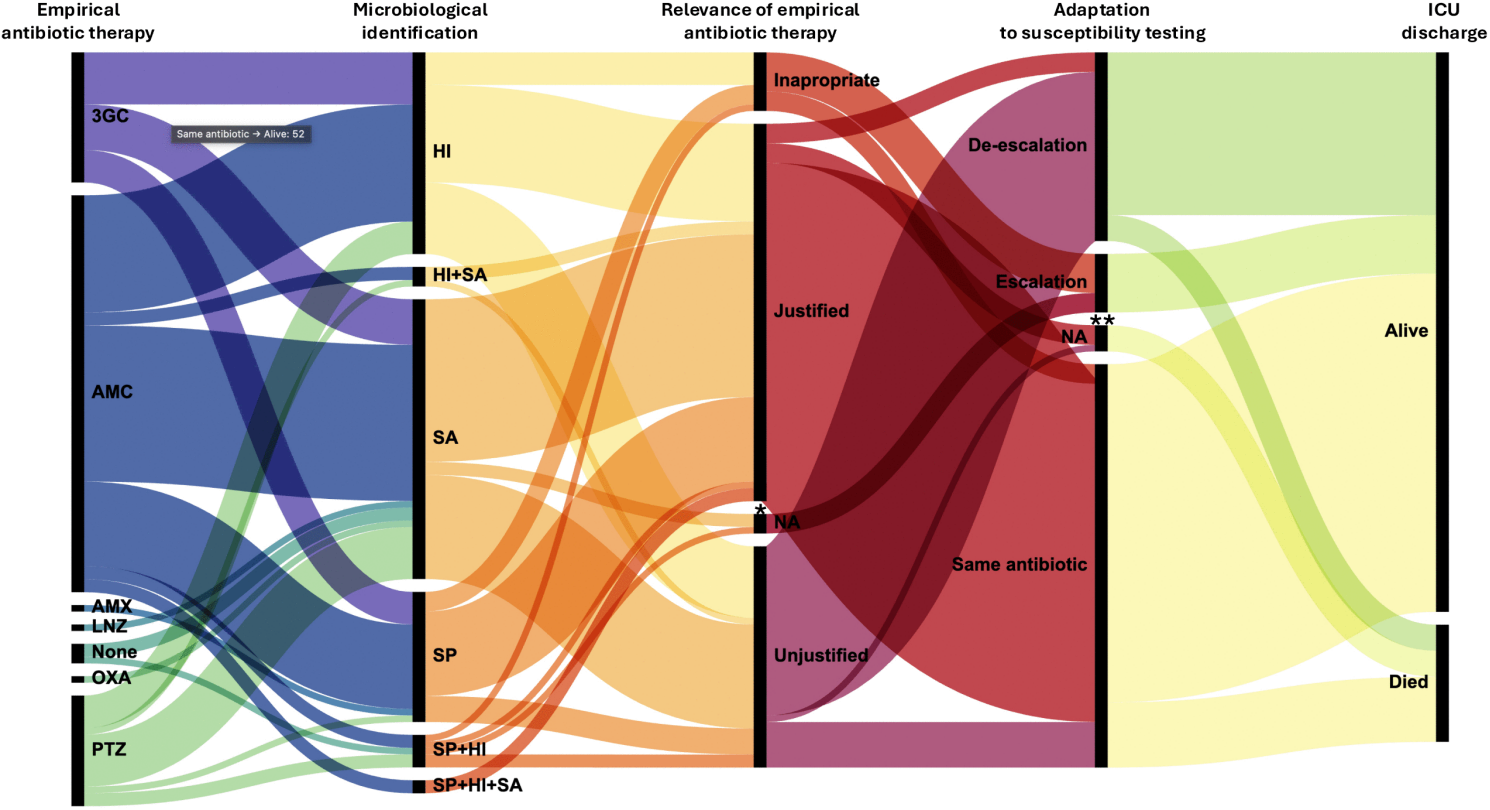
Sankey (alluvial) diagram illustrating patient pathways within the cohort, from initiation of empirical antibiotic therapy to ICU discharge. AMC: amoxicillin-clavulanate; AMX: amoxicillin; 3GC: third-generation cephalosporin; LNZ: linezolid; OXA: oxacillin; PTZ: piperacillin-tazobactam; HI: *Hæmophilus influenzæ*; SA: *Staphylococcus aureus*; SP: *Streptococcus pneumoniæ*. * Patients categorized as « NA » corresponded to the three patients who did not receive empirical antibiotic therapy; ** Patients categorized as « NA » corresponded to the four patients who died before any antibiotic adaptation.

Changes in antibiotic management and patient outcomes are illustrated in Fig 3. After availability of antimicrobial susceptibility testing results, empirical antibiotic therapy was unchanged in 62 patients (59.6%). Secondary adaptation was performed in 38 patients, including de-escalation in 29 patients (27.9%) and escalation in 9 patients (8.7%). Four patients (3.8%) died before any antibiotic adaptation could be implemented.

Details of secondary adaptations among the 98 patients who received a recommended empirical beta-lactam are presented in Table 4. Among the 61 patients treated empirically with AMC, therapy was considered appropriate in 53 cases (86.9%). In cases of inappropriate AMC use, treatment was switched to a 3GCs in 50.0% of cases, while no modification was made in 37.5%.

**Table 4:** Details of empirical beta-lactam therapies and secondary adaptation after availability of antimicrobial susceptibility testing (98 antibiotic therapies evaluated among the 104 included patients *).

Recommended beta-lactam class for CAP/ CAAP	
**Empirical AMC** ^**(a)(b)**^	**61 (100.0)**
***> Appropriate AMC***	**53 (86.9)**
***> Inappropriate AMC***	**8 (13.1)**
Switched to 3GC	4/8 (50.0)
Switched to fluoroquinolone	1/8 (12.5)
No modification	3/8 (37.5)
**Empirical 3GC** ^**(b)(c)(d)**^	**20 (100.0)**
***> Unjustified 3GC***	**16 (80.0)**
Switched to AMC	7/16 (43.8)
Switched to oxacillin	3/16 (18.7)
No modification	6/16 (37.5)
***> Justified 3GC***	**3 (15.0)**
***> Inappropriate 3GC***	**1 (5.0)**
Switched to linezolid	1/1 (100.0)
**Empirical PTZ** ^**(b)(c)**^	**17 (100.0)**
***> Unjustified PTZ***	**17 (100.0)**
Switched to AMC	8/17 (47.0)
Switched to oxacillin	5/17 (29.4)
Switched to 3GC	1/17 (5.9)
Switched to amoxicillin	1/17 (5.9)
No modification	2/17 (11.8)
***> Inappropriate PTZ***	**0 (0)**

Data are expressed as number (percentage). CAP/ CAAP: community-acquired pneumonia/ community-acquired aspiration pneumonia; AMC: amoxicillin-clavulanate; 3GC: third-generation cephalosporin; PTZ: pipéracilline-tazobactam.

* Six patients were excluded from this analysis: three patients who did not receive any documented empirical therapy and three patients treated with a non-first-line recommended antibiotic (one with amoxicillin, one with oxacillin, and one with linezolid).

**(a)** Appropriate therapy: the empirical antibiotic prescribed covered the identified pathogen based on *in vitro* susceptibility.

**(b)** Inappropriate therapy: the identified pathogen was resistant *in vitro* to the empirical antibiotic prescribed.

**(c)** Unjustified therapy (overtreatment): the empirical antibiotic prescribed covered the pathogen, but a narrower-spectrum alternative (e.g., AMC instead of a 3GC) would have been equally effective based on the susceptibility profile.

**(d)** Justified therapy: the use of a broader-spectrum agent (e.g., 3GC or PTZ) was necessary because the pathogen was resistant to narrower-sepctrum alternative (e.g., AMC).

Empirical use of 3GCs was considered unjustified in 80.0% of cases. In these situations, therapy was most often de-escalated to amoxicillin–clavulanate (43.8%) or continued without modification (37.5%). All empirical PTZ therapies were considered unjustified and were most frequently de-escalated to AMC or oxacillin.

### Clinical outcomes

No patient developed *Clostridioides difficile* infection during ICU stay. Seven patients (6.7%) acquired colonization with multidrug-resistant bacteria, including five patients in the AMC-R group. Identified organisms included ceftazidime-resistant *P. aeruginosa*, methicillin-resistant *S. aureus*, extended-spectrum beta-lactamase–producing Enterobacteriaceae, and one carbapenem-resistant Enterobacteriaceae.

Overall ICU mortality was 17.3% (18/104). All deceased patients belonged to the AMC-S group. Among these patients, empirical antibiotic therapy was considered appropriate in 13 cases (72.2%) and unjustified in 5 cases (27.8%); none received inappropriate empirical therapy.

## Discussion

The main findings of this study can be summarized as follows: 1/ Overall susceptibility to AMC remained high, reaching 86.5%. Susceptibility rates to 3GCs were nonetheless higher (97.1%); 2/ Empirical prescription of AMC, which occurred in nearly 60% of cases, was appropriate in 86.9% of situations. When AMC was used inappropriately, empirical therapy was not modified in approximately one-third of cases, without any observed negative impact on ICU survival; 3/ In contrast, 3GCs, prescribed in nearly 20% of patients, were largely used in an unjustified manner (80% of cases) and were not replaced in more than one-third of cases, without any observed impact on ICU survival.

Our findings are consistent with previous epidemiological data and confirm that AMC retains substantial *in vitro* activity against the main pyogenic bacteria responsible for severe CAP/CAAP requiring ICU admission [10,13,17]. Comparable efficacy of AMC and 3GCs has already been demonstrated in non-severe CAP [13]. However, when comparing our findings with the existing literature on severe CAP, significant heterogeneity emerges that warrants critical synthesis. In the study by Sarr et al., published in 2025 and describing 185 patients with severe CAP (including 42% of CAAP), overall susceptibility rates to AMC and 3GCs were 83.5% and 89.6%, respectively [17]. In contrast, Hariri et al., published in 2017 and including 267 patients, reported a substantially lower susceptibility rate to AMC (69.3%) [6]. This clinically meaningful difference could alter empirical prescribing decisions, and we hypothesize two main reasons for this discrepancy. First, differences in the included microbiological spectrum likely impacted the overall susceptibility profiles. While our analysis strictly focused on *S. pneumoniae*, *H. influenzae*, and *S. aureus*, the cohort by Hariri et al. also included Enterobacteriaceae (mainly *Escherichia coli* and *Klebsiella pneumoniae*) as well as non-fermenting Gram-negative bacilli (notably *P. aeruginosa*). Similarly, Sarr et al. included 18% of CAP cases caused by *E. coli* and *K. pneumoniae* (without any *P. aeruginosa*). The inclusion of these Gram-negative pathogens, which possess higher intrinsic resistance to aminopenicillins, could have inherently decreased the reported overall susceptibility to AMC. Second, the gap between 2017 and 2025 reflects a period of significant evolution in ICU practices. Over recent years, ICUs have implemented robust antimicrobial stewardship programs in close collaboration with infectious disease specialists and bacteriologists. Crucially, this era has seen the increasing and early use of multiplex polymerase chain reaction pneumonia panels, enabling rapid pathogen identification and early antibiotic adaptation [18]. In our univariate analysis, an identified *H. influenzae* infection was the sole factor associated with AMC resistance. This result is biologically plausible given that *H. influenzae* deploys multiple resistance mechanisms against aminopenicillins, notably via beta-lactamase production and structural alterations of penicillin-binding proteins [19]. However, this association should be interpreted cautiously. The restricted number of AMC-resistant patients in our cohort did not allow for a robust multivariable analysis to adjust for potential confounders. Additionally, contrasting with our data, recent literature has highlighted an improving susceptibility profile of *H. influenzae* susceptibility to AMC, including in severe pneumonia cases [10]. Regardless of these cohort-specific findings, it is crucial that well-documented risk factors for AMC resistance are systematically evaluated when selecting empirical therapy for severe CAP [6,17]. While Sarr et al. identified prior antibiotic exposure within the previous three months as the sole independent predictor for AMC resistance [17], Hariri et al. reported a complex mix of comorbidities and functional statuses [6]. As observed in our own initial exploratory analyses, this divergence highlights a major analytical limitation inherent to these specific study designs. In small, single-center retrospective cohorts, the absolute number of AMC-resistant events remain low. Attempting to build multivariable predictive models on such constrained datasets frequently leads to statistical overfitting and unstable results that reflect center-specific case mixes rather than true, universally applicable predictors. Given these glaring inconsistencies across single-center retrospective studies, clinicians cannot currently rely on a universal set of clinical predictors or scoring systems to anticipate AMC resistance in severe CAP. Consequently, empirical prescribing decisions must remain heavily driven by continuous surveillance of local ecological data. To definitively resolve this uncertainty and develop a robust, widely applicable tools for AMC resistance prediction, future research must shift away from small retrospective observations toward large-scale, prospective, multicenter cohort studies.

Despite guidelines supporting the use of 3GCs as empirical therapy for severe CAP [8], Hariri et al. reported, as in our study, a high rate of unjustified 3GCs use (65.9%), which could theoretically have been substituted by AMC in all cases. These findings therefore suggest that AMC may represent a credible alternative to 3GCs in this setting, offering a narrower antimicrobial spectrum that could help reduce antibiotic selection pressure and the emergence of multidrug-resistant bacteria [6,20–22]. Interestingly, pathogens identified as AMC–resistant in the study by Hariri et al. exhibited an overall susceptibility rate of 94% to amikacin. The authors suggested that a combination of amoxicillin–clavulanate and amikacin [6], the latter having concentration-dependent bactericidal activity and a favorable post-antibiotic effect, could also represent an alternative to empirical 3 CGs, particularly in patients with risk factors for resistance to AMC, while awaiting definitive microbiological results and subsequent antibiotic adaptation. The high proportion of monotherapy observed in our cohort may be explained by two factors. First CAAP accounted for a large proportion of cases (60%), for which AMC monotherapy remains recommended [5]. Moreover, a substantial proportion of patients presented with bacterial superinfection of viral pneumonia (17.3%), in which atypical pathogens are less frequently involved and where *S. pneumoniae*, *H. influenzae*, and *S. aureus* remain predominant [23].

### Limitation

Beyond the limitations inherent to retrospective single-center studies, several limitations should be considered when interpreting the results of this study. First, the retrospective and single-center design inherently limits the external validity of our findings. Antibiotic prescribing practices, local bacterial ecology, and resistance patterns may differ substantially across institutions and regions. As a consequence, the susceptibility rates observed in this cohort may not be directly generalizable to other ICU settings. Second, the inclusion period encompassed the SARS-CoV-2 pandemic. As detailed in the results, patients with viral co-infections, predominantly SARS-CoV-2, were not excluded provided they met the criteria for early documented bacterial CAP or CAAP, accounting for 18 patients (17.3%) in our cohort. While current literature reports early bacterial co-infections in 5–20% of severe COVID-19 cases [24–27], studies describing the specific antimicrobial susceptibility profiles of these co-infections remain scarce. This pandemic context may have transiently influenced local bacterial ecology and antibiotic prescribing strategies. However, the results of previously cited studies conducted in France confirm our findings, reporting high susceptibility rates to AMC among pathogens responsible for severe CAP and CAAP, particularly in the absence of identified risk factors for resistance to AMC [6,17]. Moreover, although antibiotic consumption increased during the SARS-CoV-2 pandemic, rates of multidrug-resistant bacteria appear to have remained stable [28]. Second, the relatively small sample size represents a major limitation, particularly for the analysis of factors associated with AMC resistance as previously acknowledged. Third, the small sample size and the very low incidence of *Clostridioides difficile* infection or colonization with multidrug-resistant bacteria in our cohort precluded evaluation of the impact of the antibiotic regimens used on the occurrence of such events. However, Razazi et al. have previously demonstrated that empirical therapy with AMC may constitute a protective factor against colonization with multidrug-resistant bacteria [29]. Fourth, we did not evaluate other pyogenic pathogens such as *Klebsiella pneumoniae*, which are less frequently implicated in severe CAP or CAAP but are associated with higher mortality, particularly in patients with chronic alcoholism, as previously reported [30]. Nevertheless, previous data suggest high susceptibility rates to beta-lactam/beta-lactamase inhibitor combinations, including AMC, in non–extended-spectrum beta-lactamase-producing strains [31], supporting the potential relevance of AMC even beyond the pathogens evaluated in this study. Finally, although no excess mortality was observed among patients receiving inappropriate empirical antibiotic therapy, these findings should not be interpreted as evidence that inappropriate therapy is without consequence. Beyond empirical antibiotic choice, several factors strongly influence patient outcomes, including the timeliness of initial management in septic shock, rapid antibiotic adaptation following microbiological documentation or clinical deterioration [32], and the susceptibility of pathogens to adjunctive therapies targeting intracellular bacteria, such as macrolides and fluoroquinolones.

## Conclusion

In this single-center retrospective cohort of ICU patients with severe CAP or CAAP due to *S. pneumoniae*, *H. influenzae*, and/or *S. aureus*, AMC showed high susceptibility rates and was frequently used appropriately, whereas 3GCs were often prescribed without microbiological justification. Given the inherent limitations of our study design, these findings cannot formally dictate an immediate shift in clinical guidelines. However, they highlight the need to question the systematic empirical use of broad-spectrum 3GCs, by conducting large-scale, randomized controlled trials to formally evaluate the clinical efficacy and safety of AMC as a first-line empirical alternative in severe CAP.

## Supporting information

S1 Table Comparison of patients’ characteristics according to amoxicillin-clavulanate susceptibility. (DOCX)

## Declarations

**Declaration of generative AI in scientific writing:** Artificial intelligence–based tools were used solely to assist with English language editing and proofreading of the manuscript. The authors take full responsibility for the scientific content, data interpretation, and conclusions presented in this article.
