# Supplementary material for: Third-generation cephalosporin use is frequently non-guideline-concordant in severe community-acquired pneumonia: Findings from a French critical care cohort
Source: PLoS One. 2026 Jul 29;21(7):e0354339. doi: 10.1371/journal.pone.0354339 (PMC13419188; doi:10.1371/journal.pone.0354339)
Supplement: S1 Table — (DOCX) [file pone.0354339.s001.docx]

**S1 Table.** **Comparison of patients’ characteristics according to amoxicillin-clavulanate susceptibility.**

| **Parameters** | **AMC-R**  **(N = 14)** | **AMC-S**  **(N = 90)** | **p value** |
| --- | --- | --- | --- |
|  |  |  |  |
| **Age** – years | 63 [37–67] | 64 [48–72] | 0.17 |
| **Female sex** | 3 (21.4) | 31 (34.4) | 0.54 |
| **Body mass index** - kg/m^2^ | 24,5 [21–29] | 25 [22–29] | 0.62 |
| **Chronic respiratory disease** | 6 (42.9) | 35 (38.9) | 0.78 |
| **Smoking history** | 9 (64.3) | 57 (65.5) | 1.00 |
| *Missing data* | *0* | *3* |  |
| **Chronic alcohol abuse** | 1 (7.1) | 23 (25.6) | 0.18 |
| **Charlson comorbidity index** | 3 [1–5] | 3 [1–5] | 0.56 |
| **SAPS II** | 44 [32–53] | 49 [35–60] | 0.29 |
| **PSI** | 133 [107–137] | 145 [120–177] | 0.06 |
| **PSI risk stratification** |  |  |  |
| Low risk (<91) | 2 (14.3) | 7 (7.8) | 0.34 |
| Moderate risk (91-130) | 5 (35.7) | 22 (24.4) |  |
| High risk (PSI >130) | 7 (50.0) | 61 (67.8) |  |
| **Antibiotic therapy before hospital admission** | 1 (7.1) | 2 (2.2) | 0.35 |
| **Antibiotic therapy within the previous 3 months** | 1 (7.7) | 3 (3.5) | 0.44 |
| *Missing data* | 1 | 3 |  |
| **Reason for ICU admission** |  |  |  |
| Respiratory | 9 (64.3) | 36 (40.0) | 0.72 |
| Neurological | 3 (21.4) | 27 (30.0) |  |
| Toxicological | 1 (7.1) | 15 (16.7) |  |
| Metabolic | 1 (7.1) | 3 (3.3) |  |
| Gastrointestinal | 0 (0) | 3 (3.3) |  |
| Cardiovascular | 0 (0) | 2 (2.2) |  |
| Trauma | 0 (0) | 1 (1.1) |  |
| Other | 0 (0) | 3 (3.3) |  |
| **Community-acquired aspiration pneumonia** | 5 (35.7) | 55 (61.1) | 0.08 |
| Continuous variables are expressed as median [interquartile range 25th-75^th^ percentile] and compared using Wilcoxon rank-sum test. Categorical variables are expressed as number (percentage) and compared using Fischer’s exact test. AMC-R: amoxicillin-clavulanate resistant; AMC-S: amoxicillin-clavulanate susceptible; SAPS II: Simplified Acute Physiology Score II; PSI: Pneumonia Severity Index (Fine score). | | | |
