# Supplementary material for: Third-generation cephalosporin use is frequently non-guideline-concordant in severe community-acquired pneumonia: Findings from a French critical care cohort
Source: PLoS One. 2026 Jul 29;21(7):e0354339. doi: 10.1371/journal.pone.0354339 (PMC13419188; doi:10.1371/journal.pone.0354339)
Supplement: Attachment — Submitted filename: Response to Reviewers.docx [file pone.0354339.s003.docx]

**RESPONSE TO REVIEWERS**

**To the Editor in Chief, to the Academic Editor, Mabel Kamweli Aworh, and to the reviewers.**

We thank again all the reviewers and the editor for their helpful and insightful comments.

Please find enclosed the new revised version of our manuscript entitled: *Third-generation cephalosporins are often unjustified in severe community-acquired pneumonia: insights from a retrospective French critical care cohort.*

All the modifications suggested by the reviewers have been fully considered. Accordingly, a new version including all the requested modifications or explanations is provided (highlighted in yellow in the Revised Manuscript with Track Changes file). Additionally, a point-by-point response including a detailed rebuttal of all comments, criticisms, and revisions has been provided separately. These responses **(R)** are enclosed below and detail all the modifications provided in the new version of the manuscript.

We hope that this last modified version will be suitable for publication in the journal. As corresponding authors, we remain at your entire disposal if any further information is requested.

Best regards.

Quitterie Lostie de Kerhorr, MD

Jonathan Chelly, MD

Journal Requirements

When submitting your revision, we need you to address these additional requirements.

**1.** Please ensure that your manuscript meets PLOS ONE's style requirements, including those for file naming.

**(R)** We have carefully reviewed and applied PLOS ONE's style requirements throughout the manuscript, including file naming conventions, formatting of the main body, title page, authors, and affiliations.

**2.** In the online submission form you indicate that your data is not available for proprietary reasons and have provided a contact point for accessing this data. Please note that your current contact point is a co-author on this manuscript. According to our Data Policy, the contact point must not be an author on the manuscript and must be an institutional contact, ideally not an individual. Please revise your data statement to a non-author institutional point of contact, such as a data access or ethics committee, and send this to us via return email. Please also include contact information for the third-party organization, and please include the full citation of where the data can be found.

**(R)** We have updated our data availability statement to comply with the journal's policy. The new institutional contact point is the *Délégation à la Recherche Clinique et à l'Innovation du Groupement Hospitalier Territorial du Var* (DRCI GHT 83). They can be contacted for data access requests at [support-recherche@drci-ght83.fr](mailto:support-recherche@drci-ght83.fr)

**3.** Your ethics statement should only appear in the Methods section of your manuscript. If your ethics statement is written in any section besides the Methods, please move it to the Methods section and delete it from any other section. Please ensure that your ethics statement is included in your manuscript, as the ethics statement entered into the online submission form will not be published alongside your manuscript.

**(R)** The ethics statement has been exclusively moved to the "Study setting and population" subsection of the Methods section. It has been completely removed from all other sections of the manuscript.

**4.** Please include captions for your Supporting Information files at the end of your manuscript, and update any in-text citations to match accordingly. Please see our Supporting Information guidelines for more information: [http://journals.plos.org/plosone/s/supporting-information](http://track.editorialmanager.com/CL0/http:%2F%2Fjournals.plos.org%2Fplosone%2Fs%2Fsupporting-information/1/010f019de255798b-4f385c9a-a24c-4a4c-aa95-4155f97b4421-000000/aTyL3K-EwxqFw559lqovOSmlCR8Yk9gI2V6XA6zPxsE=258).

**(R)** Captions for the Supporting Information files (S1 Table and S2 Table) have been appropriately added to the end of the manuscript, and all in-text citations have been updated to match this formatting.

**5.** If the reviewer comments include a recommendation to cite specific previously published works, please review and evaluate these publications to determine whether they are relevant and should be cited. There is no requirement to cite these works unless the editor has indicated otherwise. 

**(R)** We have carefully evaluated all literature mentioned by the reviewers. Relevant publications have been added to our reference list and integrated into the Discussion to enrich the scientific context of our study.

Additional Editor Comments

In addition to addressing all reviewers' comments, please move the study limitations to the last paragraph of the Discussion section. In the conclusion section, please provide some recommendations based on the key study findings.

**(R)** We thank the Editor for these structural recommendations. The study limitations have been relocated to form the final paragraph of the Discussion section. Furthermore, we have expanded the Conclusion section to include specific recommendations for clinical practice and antimicrobial stewardship based on our key findings as follows:

“In this single-center retrospective cohort of ICU patients with severe CAP or CAAP due to *S. pneumoniae*, *H. influenzae*, and/or *S. aureus*, AMC showed high susceptibility rates and was frequently used appropriately, whereas 3GCs were often prescribed without microbiological justification. Given the inherent limitations of our study design, these findings cannot formally dictate an immediate shift in clinical guidelines. However, they highlight the need to question the systematic empirical use of broad-spectrum 3GCs, by conducting large-scale, randomized controlled trials to formally evaluate the clinical efficacy and safety of AMC as a first-line empirical alternative in severe CAP.”

**Reviewer 1**

**1.** The purpose of an abstract is to summarize the key findings in an easy way to understand please rephrase the following:

“AMC was prescribed empirically in 59% of cases and was appropriate in 86.9%”. – AMC was appropriate in 86.9% of cases, however it was only prescribed empirically in 59% of those cases.

“3GCs, prescribed in 20% of cases, were mostly unjustified (80%).”

**(R)** We thank the reviewer for pointing out this lack of clarity. We have rephrased these sentences in the Abstract to make the findings more straightforward. The text has been modified to: “AMC was the empirical treatment in 59% of cases, and this choice was microbiologically appropriate in 86.9% of these prescriptions. Conversely, 3GCs were prescribed empirically in 20% of cases, but this broad-spectrum choice was unjustified in 80% of those situations."

**2. Under line 252:**

Recommended beta-lactam class for CAP/CAAP

“(a) Appropriate use of AMC was defined as susceptibility to amoxicillin and/or AMC and/or oxacillin, depending on the identified pathogen. (b) Justified use of 3GC was defined as susceptibility to 3GC with resistance to amoxicillin and/or AMC and /or oxacillin, depending on the identified pathogen. Inappropriate therapy was defined as resistance to the evaluated empirical treatment. (c) Justified use of PTZ was defined as susceptibility to PTZ with resistance to amoxicillin and/or AMC and/or oxacillin and 3GC, depending on the identified pathogen”

I am having problems with these terminologies. Appropriate for AMC was defined, inappropriate for AMC was not (could mean the opposite of what appropriate could mean for AMC). Then B we changed it to justified- also defined, but no definition for unjustified, and inappropriate means resistance to the evaluated treatment- what evaluated treatment?
Same issue for C.

**(R)** We apologize for the confusion caused by the terminology. We have thoroughly revised these definitions in the Methods and the footnotes in Table 4 to ensure clarity and consistency.

Consequently, the text was modified in the Methods section as follows:

“Prescribed empirical therapy was classified as appropriate if the antibiotic effectively covered the isolated pathogen based on susceptibility testing, and inappropriate if the pathogen was resistant to the treatment. Among appropriate prescriptions, the use of a broader-spectrum agent (3GCs or PTZ) was classified as justified if the pathogen exhibited resistance to narrower-spectrum alternatives. Conversely, it was classified as unjustified when the empirical antibiotic was active, but a narrower-spectrum recommended option would have provided identical coverage, representing a situation of overtreatment.”

And the footnotes in Table 4 were modified to:

(a) Appropriate therapy: The empirical antibiotic prescribed covered the identified pathogen based on in vitro susceptibility.
(b) Inappropriate therapy: The identified pathogen was resistant in vitro to the empirical antibiotic prescribed.
(c) Unjustified therapy (overtreatment): The empirical antibiotic prescribed covered the pathogen, but a narrower-spectrum recommended alternative (e.g., AMC instead of a 3GC) would have been equally effective based on the susceptibility profile.
(d) Justified therapy: The use of a broader-spectrum agent (e.g., 3GC or PTZ) was necessary because the pathogen was resistant to narrower-spectrum alternatives (e.g., AMC).

**Reviewer 2**

The manuscript presents a retrospective study examining the unwarranted use of third-generation cephalosporins in patients with severe community-acquired pneumonia. This research significantly contributes to antibiotic stewardship, particularly in the context of the ongoing challenge of antibiotic resistance.

It is commendable that the authors adhered to STROBE guidelines in their methodology. While the study claims to have anonymized the collected data, the manuscript lacks detail on the deidentification process (which should be addressed), given that patient information in clinical settings is often not fully deidentified.

The operationalization of variables is well presented, alongside an account of the statistical methods employed and the necessary ethical approval. Results are presented clearly, and the discussion is robust. The study's limitations are addressed with proposed further research. The references cited are relevant and well-documented, further strengthening the manuscript's credibility. Overall, the paper is written in a clear and accessible manner. The authors have communicated the unjustified use of third-generation cephalosporins in the treatment of severe community-acquired pneumonia, emphasizing the need for scrutinizing antibiotic prescribing practices.

Upon clarification of how the collected data were anonymized, this research will be suitable for publication.

**(R)** We thank the reviewer for this important methodological comment. We have added a detailed description of the de-identification process in the "Data collection and outcome variables" section. Specifically, we clarified that patient data was extracted from electronic medical records and pseudonymized assigning a unique study identification number. The correspondence key linking patient identities to their study IDs was kept on a secured, hospital-hosted server accessible only to the primary investigators. All subsequent analyses were performed strictly on the de-identified dataset.

**Reviewer 3**

I want to commend the authors for their manuscript which in the current context of AMR is clinically relevant and timely. Antibiotic stewardship in severe CAP is a genuine priority in critical care medicine, and challenging entrenched prescribing habits is valuable. I have a few comments and suggestions which could help improve the context of the manuscript and increase value for the readers.

**1. Title:**

I think the title may be a bit sensational. Severe CAP (typically ICU-level) is a specific and high-acuity population where empiric broad-spectrum coverage is often precisely the standard of care due to diagnostic uncertainty on admission. The claim that third-generation cephalosporins (3GCs) are unjustified in this population is counterintuitive and risks being misleading without immediate clarification. This makes the title sensational in a way that could undermine credibility. Suggested "Third-generation cephalosporin use is frequently non-guideline-concordant in severe community-acquired pneumonia: findings from a French critical care cohort"

**(R)** We fully agree with the reviewer that the original title could be perceived as overly definitive, especially given the necessary broad-spectrum coverage initially required in ICU settings. Consequently, we have modified the title, as suggested by the reviewer.

**2. Abstract (Lines 45 – 47):**

The authors should be mindful that this is a Single-center, retrospective design and this language needs to be stressed in the conclusion. The conclusion as it reads is kind of broadly generalizable. A single-center study of 104 patients over about 5 years in one ICU carries significant selection bias and limits external validity. This must be flagged in the conclusion, not just buried in the methodology. I suggest that you conclude by saying "In this single-center retrospective cohort, AMC appeared to be a relevant empirical option... These findings require prospective multicenter validation before practice change can be recommended."

**(R)** We thank the reviewer for his pertinent remark. We have amended the conclusion in the Abstract as suggested by the reviewer, to explicitly highlight the single-center, retrospective nature of the study, thereby avoiding overstating the external validity of our findings.

Moreover, the Conclusion section of the main manuscript was also modified in the same way, as follows:

“In this single-center retrospective cohort of ICU patients with severe CAP or CAAP due to *S. pneumoniae*, *H. influenzae*, and/or *S. aureus*, AMC showed high susceptibility rates and was frequently used appropriately, whereas 3GCs were often prescribed without microbiological justification. Given the inherent limitations of our study design, these findings cannot formally dictate an immediate shift in clinical guidelines. However, they highlight the need to question the systematic empirical use of broad-spectrum 3GCs, by conducting large-scale, randomized controlled trials to formally evaluate the clinical efficacy and safety of AMC as a first-line empirical alternative in severe pneumonia.”

**3. Abstract (Lines 30 – 33):**

Your abstract does not mention the AMC resistance risk factor objective mentioned in your main manuscript. This is a significant inconsistency between your abstract and manuscript body that must be corrected.

**(R)** We thank the reviewer for raising this point. While we acknowledge that evaluating risk factors for AMC resistance was a secondary objective of our study, we deliberately chose not to include this specific analysis in the abstract for two main reasons. First, the strict 300-word limit imposed required us to prioritize our primary descriptive findings regarding overall susceptibility rates and the appropriateness of empirical prescriptions. Second, and most importantly, as we explicitly acknowledge in the manuscript, the limited sample size (specifically the low number of AMC-resistant events; n=14) considerably restricts the statistical power and robustness of our multivariable model. We felt that featuring this exploratory finding in the abstract might overstate its validity and potentially mislead the reader by presenting it as a definitive conclusion. Therefore, we prefer to detail this finding exclusively within the Results and Discussion sections of the main text, where its inherent limitations can be properly and fully contextualized. We hope the reviewer will understand and agree with this editorial and methodological choice.

**4. Abstract (Lines 38 – 43):**

Though the sample size is small, but confidence intervals are absent. Having 104 patients over nearly 5 years is a modest cohort. Susceptibility rates reported as point estimates (e.g., 81.5% for SP) with no confidence intervals are insufficient. Please add CIs to all primary endpoint results.

(R) We thank the reviewer for this pertinent statistical reminder. We completely agree that reporting point estimates of proportions without uncertainty measures is insufficient, particularly given our sample size. We have now calculated and added 95% Confidence Intervals (95% CIs) to all primary susceptibility outcomes (proportions) in the Abstract, the Results section, and the relevant figures and tables. For instance, the AMC susceptibility rate for *S. pneumoniae* is now correctly reported as 81.5% [95%CI 61.9–93.7]. Furthermore, we have updated the "Statistical analysis" section to state that these confidence intervals were calculated using the exact binomial method (Clopper-Pearson), which is the most robust approach for small sample sizes.

**5. Introduction (Lines 67 – 70):**

The authors need to provide a clear statement in the introduction on the distinction between severity of CAP. When is CAP said to be severe? Because up to this point in the introduction, the topic was just on CAP. It is crucial to state/justify "that severe CAP is distinct from non-severe CAP and warrants separate consideration" before now going to talk about the bacteria causing CAP.

**(R)** We thank the reviewer for this insightful comment. We agree that the distinction between non-severe and severe CAP were insufficiently emphasized. We have revised the Introduction section as follows:

“CAP is defined as severe when ICU admission is required, most frequently due to acute respiratory failure requiring mechanical ventilation (MV) and/or the presence of septic shock requiring vasopressors. It is crucial to distinguish severe CAP from non-severe forms, as it warrants separate therapeutic consideration. Indeed, the high immediate mortality risk associated with severe CAP (reaching up to 30% in patients under MV) **[1,4]** dictates the need for prompt and often broader empirical antibiotic therapy upon admission, differing substantially from standard medical ward protocols.”

**6. Introduction (Lines 67 – 70):**

Though the authors made a brief mention of CAAP as a clinically subgroup of CAP, it is not immediately clear or justified why it is included alongside CAP is it because of shared pathogens? overlapping management? this must be justified clearly not implied.

**(R)** We thank the reviewer for pointing out this lack of explicit justification. We have updated the Introduction to state the rationale for including CAAP alongside CAP in our analysis, as follows:

“CAAP is frequently evaluated alongside severe CAP in the ICU setting. This grouping is justified because both conditions present with overlapping acute respiratory failure, are often difficult to reliably distinguish upon initial admission, and are managed under similar initial severity protocols.”

**7. Methods (Lines 99 – 115):**

It’s important to provide a bit more context concerning the study center to better contextualize the area and get better interpretation of results. Include crucial information on type of ICU (medical, mixed), number of beds, academic vs. community, tertiary referral status. Describe the ICU case volume relevant to pneumonia, this contextualizes whether 104 patients over ~5 years is expected or surprisingly low.

Can you justify the endpoint of your study? (e.g., change in local guidelines, COVID-19 impact, formulary change) why did you decide to study just these years?

**(R)** We thank the reviewer for this excellent suggestion, which greatly improves the contextualization of our study.

First, we have expanded the "Study setting and population" section to include crucial details about our center. We specified that the study took place in the mixed medical-surgical ICU of a non-academic tertiary referral hospital, equipped with 16 ICU beds and 8 intermediate care beds. We also justified the study period in the Methods section, explaining that this period was chosen to capture a stable picture of contemporary bacterial ecology and empirical prescribing habits just prior to the transition toward new national guidelines and the implementation of updated local antimicrobial stewardship protocols.

Second, regarding the reviewer's legitimate question about the case volume (whether 104 patients over 5 years is expected or surprisingly low), we would like to clarify that a total of 286 patients were admitted to our ICU with a considered severe CAP/CAAP during the study period. This equates to roughly 60 severe pneumonia admissions per year, which is a consistent and standard volume such ICU. As already detailed in our Results section and the study flow chart (Fig 1), the final cohort size of 104 patients is the direct consequence of our stringent inclusion criteria, which strictly required definitive microbiological documentation with the considered pathogens with available susceptibility testing.

**8. Study period:**

There is need to address the COVID-19 overlap (2020–2022) must be addressed. Did you exclude COVID-19 pneumonia? Did the pandemic alter your ICU's antibiotic prescribing practices or microbiological documentation rates? These are major issues that needs to be addressed and that add to the context surrounding your study.

**(R)** We thank the reviewer for raising this important point regarding the COVID-19 overlap. To clarify our methodology, patients with COVID-19 pneumonia were not excluded from our study, provided they presented with an early, microbiologically documented bacterial co-infection (*S. pneumoniae*, *H. influenzae*, and/or *S. aureus*) upon ICU admission. As reported in our Results section, 18 patients (17.3%) in our cohort had a documented viral co-infection, predominantly SARS-CoV-2. We have significantly expanded the Discussion section to address the pandemic's potential impact. We acknowledge that while the literature estimates bacterial co-infection rates in severe COVID-19 to be between 5% and 20%, detailed data regarding the antimicrobial susceptibility profiles of these specific early co-infections remain extremely scarce. Most available studies on this specific topic originate from Middle Eastern cohorts, which limits our ability to make robust epidemiological comparisons with Western European ICU settings. Finally, we maintained our reference to recent national data demonstrating that, despite the undeniable increase in overall antibiotic consumption during the pandemic waves in French ICUs, the overall incidence rates of multidrug-resistant bacteria remained remarkably stable.

To fully support these new statements and enrich the scientific context of this discussion, we have implemented 4 new references in the revised manuscript, as follows:

[24] Elabbadi A, Turpin M, Gerotziafas G, Teulier M, Voiriot G, Fartoukh M. Bacterial coinfection in critically ill COVID-19 patients with severe pneumonia. Infection 2021;49:559–62.

[25] Moolla M, Reddy K, Fwemba I, Nyasulu P, Taljaard J, Parker A et al. Bacterial infection, antibiotic use and COVID-19: Lessons from the intensive care unit. S Afr Med J 2021;111:575–81.

[26] Bergmann F, Gabler C, Nussbaumer-Pröll A, Wölfl-Duchek M, Blaschke A, Radtke C et al. Early bacterial coinfections in patients admitted to the ICU with COVID-19 or Influenza: A retrospective cohort study. Critical Care Explorations 2023;5:e0895.

[27] Rouzé A, Martín-Loeches I, Póvoa P, Metzelard M, Du Cheyron D, Lambiotte F et al. Early bacterial identification among intubated patients with COVID-19 or Influenza pneumonia: A european multicenter comparative clinical trial. Am J Respir Crit Care Med 2021;204:546–56.

**9. Lines 104 - 109:**

I think you need to make a good case for your exclusion criteria. How did you ensure you excluded all patients with healthcare-associated pneumonia (HCAP)? did you exclude patients with nursing home residence, dialysis, or home IV therapy?

Also, immunocompromised patients i.e. transplant recipients, HIV, chemotherapy etc. did you include or exclude? This affects pathogen spectrum dramatically. How did you deal with COVID-19 pneumonia? This is critical given your timeframe. And polymicrobial infections with non-targeted pathogens how were these handled? How did you handle incomplete microbiological or clinical records? These are critical areas you need to describe for your exclusion criteria.

**(R)** We thank the reviewer for requesting these crucial clarifications regarding our exclusion criteria, which help to demonstrate the homogeneity of our study population. We have addressed each sub-question below:

- Patients presenting with HCAP were excluded during the screening process. We have now specified in the text that this exclusion explicitly encompassed nursing home residents and patients receiving home intravenous (IV) therapy. Please not that patients undergoing chronic dialysis were not systematically excluded from our cohort, provided they had not been hospitalized for any reason within the three months preceding their admission.

- Immunocompromised patients (such as transplant recipients, HIV, or those receiving active chemotherapy) were excluded *by definition* from our cohort, as they do not fulfill the standard diagnostic criteria for CAP or CAAP. Consequently, they were not even evaluated among the initial pool of eligible patients.

- As detailed above, patients with COVID-19 pneumonia were not included unless they presented with an early documented bacterial co-infection matching our target pathogens upon ICU admission.

- Regarding polymicrobial cases, only infections involving strictly the targeted pathogens (*S. pneumoniae*, and/or *H. influenzae*, and/or *S. aureus*) were eligible for inclusion. As illustrated in our study flow chart (Fig 1), any polymicrobial infection involving non-targeted pathogens was systematically excluded.

- Incomplete Records: Patients with missing or incomplete clinical or microbiological records were also systematically excluded, as detailed in Figure 1.

Accordingly, we have revised the "Study setting and population" subsection to incorporate all these details, as follows:

“Patients hospitalized within the three months preceding admission, patients with another documented infection within 48 hours after admission, patients with late microbiological sampling (>48 hours), and patients who died or were discharged before bacteriological documentation were excluded. Ventilator-associated pneumonia and healthcare-associated pneumonia (including nursing home residents or patients receiving home intravenous therapy) were also excluded. Immunocompromised patients (such as transplant recipients, patients with HIV, or those undergoing active chemotherapy) were excluded by definition, as they do not meet the diagnostic criteria for CAP or CAAP, and were therefore not evaluated during the initial screening. Regarding polymicrobial infections, only cases involving the targeted pathogens were included. Finally, patients admitted for organ donation, those under legal protections measures, or those with incomplete microbiological or clinical records were excluded.”

**10. Line 104:**

What clinical/radiological criteria distinguished CAAP from CAP? It is not clear how you distinguished these 2 from the data. Did you use a validated definition or an institutional one? Were CAP and CAAP managed under the same empirical antibiotic protocol or different ones? Justify why they are analyzed together or report whether you did a subgroup analysis separating them. These should be clearly stated in your methods section but are missing

**(R)** We thank the reviewer for requesting these clarifications regarding the distinction and management of CAP and CAAP in our cohort.

CAAP was distinguished from CAP based on the clinical judgment of the attending intensive care physicians, in accordance with the diagnostic criteria outlined in the recent French guidelines (listed as reference 5). CAAP is considered a specific clinical entity integrated within the broader spectrum of CAP.

Moreover, it is crucial to emphasize that, considering the specific pyogenic bacteria targeted in our study (*S. pneumoniae*, *H. influenzae*, and *S. aureus*), the empirical antibiotic strategies for severe CAAP are identical to those recommended for severe CAP. Both conditions were therefore managed under the exact same empirical antibiotic protocols in our ICU.

Finally, this shared initial severity, combined with identical empirical treatment strategies for our targeted pathogens, supports our rationale for analyzing them together. To make this justification completely transparent to the reader, we have explicitly added the following clarification to the Introduction:

"CAAP is frequently evaluated alongside severe CAP in the ICU setting. This grouping is justified because both conditions present with overlapping acute respiratory failure, are often difficult to reliably distinguish upon initial admission, and are managed under similar initial severity protocols."

Furthermore, to provide the granularity requested, we have separated the susceptibility profiles of CAP and CAAP in the revised Figure 2, as detailed in our response to your specific comment regarding the figures.

**11. Lines 140 - 142:**

For the sake of reproducibility, how did you define appropriateness? Appropriate = at least one prescribed antibiotic covers all documented pathogens based on in vitro susceptibility? Or based on guideline concordance? Or both? And who adjudicated appropriateness? Clinician, pharmacist, independent reviewer? This information is crucial for your methods section.

**(R)** We thank the reviewer for highlighting the need for this methodological clarification. We have expanded on the definition and adjudication of appropriateness in the Methods section. Appropriateness was strictly defined based on *in vitro* susceptibility matching (i.e., at least one prescribed empirical beta-lactam effectively covered the documented pathogen). The antimicrobial susceptibility profiles were provided by our bacteriology laboratory with systematic validation by a medical bacteriologist. The final adjudication of whether the empirical therapy was "appropriate," "inappropriate," or "unjustified" was performed retrospectively by the investigators, based on a consensus agreement between a medical bacteriologist and an intensive care physician. We have updated the Methods section to include these important details, as follows:

“All susceptibility profiles were systematically validated by a medical bacteriologist. The clinical relevance of the empirical beta-lactam therapy was evaluated retrospectively by the investigators, based on consensus agreement between a medical bacteriologist and an ICU physician.”

**12 and 13.**

**Lines 145 - 154:** Which goodness-of-fit measure was used? Hosmer-Lemeshow test? Nagelkerke R²? AUC/c-statistic? All of the above? Include this in the analysis section of the methods. Also, there is no mention of missing data here. Missing data handling is a major source of bias in retrospective studies and must be explicitly addressed.

**Lines 148 - 149:** Your multivariate model building is not completely described. Though the p<0.20 threshold for variable entry is stated, but several elements are missing:

- Maximum number of variables allowed in the model given sample size

- The standard rule of thumb is 1 variable per 10 events (EPV rule)

With n=104 and presumably modest AMC resistance rates, your event count may be very limited. This should be stated

- How did you handle collinear variables were handled at model entry?

- Was backward, forward, or enter selection used after univariate screening?

- Were interaction terms considered?

Please clarify all of these issues.

**(R)** We thank the reviewer for these relevant statistical queries, which prompted us to critically re-evaluate the robustness of our multivariable model. Upon methodological review, we acknowledged a significant limitation: with only 14 events (AMC-resistant cases) in our cohort, performing a multivariable logistic regression with multiple clinically relevant predictors severely violates the standard EPV rule. Adjusting for only 1 or 2 variables to satisfy this rule would lack clinical meaning, while keeping all relevant variables exposes the model to severe overfitting and unreliable confidence intervals. Therefore, to maintain the statistical rigor and avoid drawing misleading conclusions from an underpowered model, we have decided to remove the multivariable analysis from the revised manuscript (including S2 Table). We believe this decision significantly strengthens the overall scientific integrity of the paper.

We have also modified the discussion section regarding our results as follows:

“In our univariate analysis, an identified *H. influenzae* infection was the sole factor associated with AMC resistance. This result is biologically plausible given that *H. influenzae* deploys multiple resistance mechanisms against aminopenicillins, notably via beta-lactamase production and structural alterations of penicillin-binding proteins **[18]**. However, this association should be interpreted cautiously. The restricted number of AMC-resistant patients in our cohort did not allow for a robust multivariable analysis to adjust for potential confounders. Additionally, contrasting with our data, recent literature has highlighted an improving susceptibility profile of *H. influenzae* susceptibility to AMC, including in severe pneumonia cases **[10]**. Regardless of these cohort-specific findings, it is crucial that well-documented risk factors for AMC resistance are systematically evaluated when selecting empirical therapy for severe CAP **[6,17]**.”

**14. Lines 146 - 147:**

You correctly use non-parametric tests (Wilcoxon rank-sum) and report medians with IQR, this implies non-normal distribution. However, was normality formally tested (Shapiro-Wilk) or assumed based on visual inspection (Q-Q plots, histograms)?

It is important you state the basis for choosing non-parametric tests

**(R)** We thank the reviewer for this remark. We opted to assess normality through a visual inspection of histograms rather than relying on formal statistical tests. We have updated the Statistical Analysis section to provide this clarification.

**15. Line 153:**

The R++ is not a conventional software used for this type of data analysis but is still acceptable. (I am not familiar with this software) May I know the justification for using this software (just curious)

**(R)** We thank the reviewer for his curiosity. This software was chosen because it combines the statistical reliability and methodological rigor of R with a user-friendly interface specifically designed for domain experts. The software is built on top of R and relies on established R statistical procedures. It also providing an environment that is accessible to clinicians and researchers.

**16. Figure 2 has some issues namely:**

A. Axes have no labels. Also, no Absolute Numbers (n) on the Bars or Axes. Percentages alone are misleading without denominators S. aureus showing 100% susceptibility to both agents looks identical to a finding based on 3 isolates vs. 30 isolates which the reader cannot tell. This is especially critical for the overall cohort bar where patients may have multiple pathogens. Add n= below each species name on the x-axis:

e.g., Streptococcus pneumoniae (n=27) or include in the bar itself

B. No Error Bars or Confidence Intervals. For a primary endpoint in this study, point estimates without uncertainty measures are insufficient. The 81.5% AMC susceptibility for SP with no CI could represent a very wide range given small n. Either add 95% CI error bars to each segment, or present CIs in an accompanying table and reference it in the figure legend

C. Your abstract and methods use "3GC" (third-generation cephalosporins) while the figure uses "C3G" please be consistent.

D. The "Overall Cohort" Column Is Statistically Problematic as patients with co-infections (e.g., SP + HI) contribute isolates to multiple species columns AND to the overall cohort column. This means the overall cohort bar does not represent independent observations. It is unclear whether the overall bar represents: % of isolates that were susceptible (isolate-level analysis), % of patients whose empirical therapy was covered (patient-level analysis)
These are fundamentally different metrics and must be distinguished.

E. CAP vs. CAAP Breakdown Is Absent: Given that 57.7% of your cohort had CAAP and SA is likely overrepresented there, susceptibility profiles may differ meaningfully between CAP and CAAP. A single merged figure obscures this potentially important finding. You could split into two panels: Panel A = CAP | Panel B = CAAP or at minimum, acknowledge in the legend that CAP and CAAP are combine. Either remove the overall cohort bar and replace with a patient-level appropriateness figure or add a clear footnote explaining the unit of analysis and how co-infections were handled.

**(R)** We thank the reviewer for all these detailed suggestions to improve Figure 2. We fully agree with all the points raised. To thoroughly address each of your concerns, we have completely redesigned Figure 2 into a comprehensive, high-resolution 3-panel layout:

- We have added clear Y-axis labels ("Susceptibility Rate %") and explicitly provided the absolute sample sizes (N) below every single bar to ensure full transparency. Furthermore, we have included 95% CI error bars for each proportion, as already stated, illustrating the statistical uncertainty of our limited sample sizes.

- We have standardized the abbreviation to "3GC-S" across all panels, as well as in the main text and tables.

- Panel A now explicitly separates the data into two distinct analyses: an isolate-level profile (treating the 116 positive samples independently) and a patient-level profile (N=104 patients), which accounts for the polymicrobial combinations to accurately reflect the adequacy of empirical therapy choices.

- To provide the requested clinical granularity, the new figure fully segregates the cohort. Panel B now displays the isolate-level results strictly for standard CAP, and Panel C strictly for CAAP.

**17. Lines 256 - 356:**

The discussion appears to be merely descriptive rather than a critical synthesis that attempts to explain the heterogeneity across studies. So, the discussion cites Sarr et al. (2025) and Hariri et al. (2017) but never critically reconciles the discrepancies between their findings and yours.

Hariri et al. reported AMC susceptibility of 69.3% — substantially lower than your 86.5% and Sarr et al.'s 83.5%. This is a clinically meaningful difference of about 17 percentage points that could change empirical prescribing decisions. The discussion acknowledges the difference exists but does not explain it. Similarly, the independent risk factors for AMC resistance differ across all three studies: Your study: HI infection only, Sarr et al.: prior antibiotic exposure only, Hariri et al.: chronic respiratory disease, cancer, prior stroke, functional autonomy, prior antibiotic exposure. The discussion presents these as parallel findings without asking why they diverge. This is a significant analytical gap. Please build a critical synthesis that attempts to explain the heterogeneity across studies, acknowledges what remains unresolved, and explicitly states what this means for clinical practice and future research. The discussion should answer: "Given these inconsistencies across three single-center retrospective studies, what can clinicians actually conclude, and what study design would resolve the uncertainty?"

**(R)** We thank the reviewer who correctly point out a significant analytical gap in our previous draft. We fully agree that merely listing parallel findings without explaining their divergence limits the clinical impact of our paper.

Accordingly, and in line with our decision to remove our own underpowered multivariable analysis (as discussed above), we have added a dedicated paragraph in the Discussion to critically synthesize these discrepancies.

First, to explain the significant difference in AMC susceptibility between Hariri et al. (2017) and recent cohorts (ours and Sarr et al.), we have introduced two main hypotheses in the revised text:

- We explicitly point out that unlike our cohort, which strictly focused on *S. pneumoniae*, *H. influenzae*, and *S. aureus*, the cohorts by Hariri et al. and Sarr et al. included Enterobacteriaceae (*E. coli*, *K. pneumoniae*) and, in Hariri's case, non-fermenting Gram-negative bacilli (*P. aeruginosa*). The inclusion of pathogens with higher intrinsic resistance inevitably lowers the reported overall AMC susceptibility.

- We highlight that the gap between 2017 and 2025 reflects a major era of evolution in antimicrobial stewardship, notably driven by the increasing and early use of multiplex PCR pneumonia panels, which we now specifically (new reference 18. de Albuquerque Pesoa Dos Santos Y, Tomazini BM, Claro Dos Santos MH, Lyra de Queiroz E, Pastore Junior L, Leite Vieira Costa E et al. Impact of syndromic molecular diagnostics on antimicrobial adequacy and tome to therapy in critically ill patients with pneumonia: a systematic review and meta-analysis of randomized trials. Critical Care 2025;29:379. https://doi.org/10.1186/s13054-025-05623-0)

Second, we explicitly address the profound divergence in independent risk factors across the three studies. We linked this inconsistency to the inherent limitations of small, single-center retrospective cohorts. As we acknowledged regarding our own initial model (see above), attempting to build multivariable predictive models with a low absolute number of AMC-resistant events inevitably leads to statistical overfitting. This produces unstable results that reflect center-specific case mixes rather than universally applicable predictors.

Finally, we directly answered your concluding questions within the manuscript: we stated that clinicians cannot currently rely on universal clinical predictors for AMC resistance but must depend on continuous surveillance of local ecological data. Furthermore, we concluded that large-scale, prospective, multicenter cohort studies are the only viable design to definitively resolve this uncertainty.
