# Supplementary material for: Third-generation cephalosporin use is frequently non-guideline-concordant in severe community-acquired pneumonia: Findings from a French critical care cohort
Source: PLoS One. 2026 Jul 29;21(7):e0354339. doi: 10.1371/journal.pone.0354339 (PMC13419188; doi:10.1371/journal.pone.0354339)
Supplement: Peer review history

# Peer review: Decision Letter 0

**DOI:** 10.1371/journal.pone.0354339.r001

**Authors:** Mabel Aworh

1 May 2026

PONE-D-26-15039

Third-generation cephalosporins are often unjustified in severe community-acquired pneumonia: insights from a retrospective French critical care cohort.

PLOS One

Dear Dr.  Chelly,

Thank you for submitting your manuscript to PLOS ONE. After careful consideration, we feel that it has merit but does not fully meet PLOS ONE’s publication criteria as it currently stands. Therefore, we invite you to submit a revised version of the manuscript that addresses the points raised during the review process.

Please submit your revised manuscript by Jun 15 2026 11:59PM. If you will need more time than this to complete your revisions, please reply to this message or contact the journal office at plosone@plos.org. When you're ready to submit your revision, log on to https://www.editorialmanager.com/pone/ and select the 'Submissions Needing Revision' folder to locate your manuscript file.

Please include the following items when submitting your revised manuscript:

- A letter that responds to each point raised by the academic editor and reviewer(s). You should upload this letter as a separate file labeled 'Response to Reviewers'.
- A marked-up copy of your manuscript that highlights changes made to the original version. You should upload this as a separate file labeled 'Revised Manuscript with Track Changes'.
- An unmarked version of your revised paper without tracked changes. You should upload this as a separate file labeled 'Manuscript'.

If you would like to make changes to your financial disclosure, please include your updated statement in your cover letter. Guidelines for resubmitting your figure files are available below the reviewer comments at the end of this letter.

If applicable, we recommend that you deposit your laboratory protocols in protocols.io to enhance the reproducibility of your results. Protocols.io assigns your protocol its own identifier (DOI) so that it can be cited independently in the future. For instructions see: https://journals.plos.org/plosone/s/submission-guidelines#loc-laboratory-protocols. Additionally, PLOS ONE offers an option for publishing peer-reviewed Lab Protocol articles, which describe protocols hosted on protocols.io. Read more information on sharing protocols at https://plos.org/protocols?utm_medium=editorial-email&utm_source=authorletters&utm_campaign=protocols.

As the corresponding author, your ORCID iD is verified in the submission system and will appear in the published article. PLOS supports the use of ORCID, and we encourage all coauthors to register for an ORCID iD and use it as well. Please encourage your coauthors to verify their ORCID iD within the submission system before final acceptance, as unverified ORCID iDs will not appear in the published article. *Only*  the individual author can complete the verification step; PLOS staff *cannot*  verify ORCID iDs on behalf of authors.

We look forward to receiving your revised manuscript.

Kind regards,

Mabel Kamweli Aworh, DVM, MPH, PhD. FCVSN

Academic Editor

PLOS One

Journal Requirements:

When submitting your revision, we need you to address these additional requirements.

1.Please ensure that your manuscript meets PLOS ONE's style requirements, including those for file naming. The PLOS ONE style templates can be found at

https://journals.plos.org/plosone/s/file?id=wjVg/PLOSOne_formatting_sample_main_body.pdf and

https://journals.plos.org/plosone/s/file?id=ba62/PLOSOne_formatting_sample_title_authors_affiliations.pdf

2. In the online submission form you indicate that your data is not available for proprietary reasons and have provided a contact point for accessing this data. Please note that your current contact point is a co-author on this manuscript. According to our Data Policy, the contact point must not be an author on the manuscript and must be an institutional contact, ideally not an individual. Please revise your data statement to a non-author institutional point of contact, such as a data access or ethics committee, and send this to us via return email. Please also include contact information for the third party organization, and please include the full citation of where the data can be found.

3. Your ethics statement should only appear in the Methods section of your manuscript. If your ethics statement is written in any section besides the Methods, please move it to the Methods section and delete it from any other section. Please ensure that your ethics statement is included in your manuscript, as the ethics statement entered into the online submission form will not be published alongside your manuscript.

4. Please include captions for your Supporting Information files at the end of your manuscript, and update any in-text citations to match accordingly. Please see our Supporting Information guidelines for more information: http://journals.plos.org/plosone/s/supporting-information.

5. If the reviewer comments include a recommendation to cite specific previously published works, please review and evaluate these publications to determine whether they are relevant and should be cited. There is no requirement to cite these works unless the editor has indicated otherwise.

Additional Editor Comments:

In addition to addressing all reviewers' comments, please move the study limitations to the last paragraph of the Discussion section. In the conclusion section, please provide some recommendations based on the key study findings.

[Note: HTML markup is below. Please do not edit.]

Reviewer's Responses to Questions

**Comments to the Author**

1. Is the manuscript technically sound, and do the data support the conclusions?

The manuscript must describe a technically sound piece of scientific research with data that supports the conclusions. Experiments must have been conducted rigorously, with appropriate controls, replication, and sample sizes. The conclusions must be drawn appropriately based on the data presented.

Reviewer #1: Yes

Reviewer #2: Yes

Reviewer #3: Yes

**********

2. Has the statistical analysis been performed appropriately and rigorously?

Reviewer #1: Yes

Reviewer #2: Yes

Reviewer #3: No

**********

3. Have the authors made all data underlying the findings in their manuscript fully available?

The PLOS Data policy requires authors to make all data underlying the findings described in their manuscript fully available without restriction, with rare exception (please refer to the Data Availability Statement in the manuscript PDF file). The data should be provided as part of the manuscript or its supporting information, or deposited to a public repository. For example, in addition to summary statistics, the data points behind means, medians and variance measures should be available. If there are restrictions on publicly sharing data—e.g. participant privacy or use of data from a third party—those must be specified.

Reviewer #1: Yes

Reviewer #2: Yes

Reviewer #3: Yes

**********

4. Is the manuscript presented in an intelligible fashion and written in standard English?

PLOS ONE does not copyedit accepted manuscripts, so the language in submitted articles must be clear, correct, and unambiguous. Any typographical or grammatical errors should be corrected at revision, so please note any specific errors here.

Reviewer #1: Yes

Reviewer #2: Yes

Reviewer #3: Yes

**********

5. Review Comments to the Author

Please use the space provided to explain your answers to the questions above. You may also include additional comments for the author, including concerns about dual publication, research ethics, or publication ethics. (Please upload your review as an attachment if it exceeds 20,000 characters)

Reviewer #1: The purpose of an abstract is to summarize the key findings in an easy way to understand please rephrase the following:

“AMC was prescribed empirically in 59% of cases and was appropriate in 86.9%”. – Amc was appropriate in 86.9% of cases, however it was only prescribed empirically in 59% of those cases.

“3GCs, prescribed in 20% of cases, were mostly unjustified (80%).”

Under line 252:

Recommended beta-lactam class for CAP/CAAP

“(a) Appropriate use of AMC was defined as susceptibility to amoxicillin and/or AMC and/or oxacillin, depending on the identified pathogen. (b) Justified use of 3GC was defined as susceptibility to 3GC with resistance to amoxicillin and/or AMC and /or oxacillin, depending on the identified pathogen. Inappropriate therapy was defined as resistance to the evaluated empirical treatment. (c) Justified use of PTZ was defined as susceptibility to PTZ with resistance to amoxicillin and/or AMC and/or oxacillin and 3GC, depending on the identified pathogen”

I am having problems with these terminologies

Appropriate for AMC was defined, inappropriate for AMC was not (could mean the opposite of what appropriate could mean for AMC).

Then B we changed it to justified- also defined, but no definition for unjustified, and inappropriate means resistance to the evaluated treatment- what evaluated treatment?

Same issue for C.

Reviewer #2: The manuscript presents a retrospective study examining the unwarranted use of third-generation cephalosporins in patients with severe community-acquired pneumonia. This research significantly contributes to antibiotic stewardship, particularly in the context of the ongoing challenge of antibiotic resistance.

It is commendable that the authors adhered to STROBE guidelines in their methodology. While the study claims to have anonymized the collected data, the manuscript lacks detail on the deidentification process (which should be addressed), given that patient information in clinical settings is often not fully deidentified. The operationalization of variables is well presented, alongside an account of the statistical methods employed and the necessary ethical approval.

Results are presented clearly, and the discussion is robust. The study's limitations are addressed with a proposed further research. The references cited are relevant and well-documented, further strengthening the manuscript's credibility.

Overall, the paper is written in a clear and accessible manner. The authors have communicated the unjustified use of third-generation cephalosporins in the treatment of severe community-acquired pneumonia, emphasizing the need for scrutinizing antibiotic prescribing practices. Upon clarification of how the collected data were anonymized, this research will be suitable for publication.

Reviewer #3: I want to commend the authors for their manuscript which in the current context of AMR is clinically relevant and timely. Antibiotic stewardship in severe CAP is a genuine priority in critical care medicine, and challenging entrenched prescribing habits is valuable. I have a few comments and suggestions which could help improve the context of the manuscript and increase value for the readers.

1. Title: I think the title may be a bit sensational. Severe CAP (typically ICU-level) is a specific and high-acuity population where empiric broad-spectrum coverage is often precisely the standard of care due to diagnostic uncertainty on admission. The claim that third-generation cephalosporins (3GCs) are unjustified in this population is counterintuitive and risks being misleading without immediate clarification. This makes the title sensational in a way that could undermine credibility. Suggested "Third-generation cephalosporin use is frequently non-guideline-concordant in severe community-acquired pneumonia: findings from a French critical care cohort"

2. Abstract: Lines 45 - 47 The authors should be mindful that this is a Single-center, retrospective design and this language needs to be stressed in the conclusion. The conclusion as it reads is kind of broadly generalizable. A single-center study of 104 patients over about 5 years in one ICU carries significant selection bias and limits external validity. This must be flagged in the conclusion, not just buried in the methodology. I suggest that you conclude by saying "In this single-center retrospective cohort, AMC appeared to be a relevant empirical option... These findings require prospective multicenter validation before practice change can be recommended."

3. Abstract: Lines 30 - 33:Your abstract does not mention the AMC resistance risk factor objective mentioned in your main manuscript. This is a significant inconsistency between your abstract and manuscript body that must be corrected.

4. Abstract: Lines 38 - 43: Though the sample size is small but confidence intervals are absent. Having 104 patients over nearly 5 years is a modest cohort. Susceptibility rates reported as point estimates (e.g., 81.5% for SP) with no confidence intervals are insufficient. Please add CIs to all primary endpoint results.

4. Introduction: Lines 67 - 70: The authors need to provide a clear statement in the introduction on the disticntion between svevrity of CAP. When is CAP said to be severe? Because up to this point in the introduction, the topic was just on CAP. It is crucial to state/justify "that severe CAP is distinct from non-severe CAP and warrants separate consideration" before now going to talk about the bacteria causing CAP.

5. Introduction: Lines 67 - 70: Though the authors made a brief mention of CAAP as a clinically subgroup of CAP, it is not immediately clear or justified why it is included alongside CAP is it because of shared pathogens? overlapping management? this must be justified clearly not implied

6. Methods Lines 99 - 115: Its important to provide a bit more context concerning the study center to better contextualize the area and get better interpretation of results. Include crucial information on type of ICU (medical, mixed), number of beds, academic vs. community, tertiary referral status. Describe the ICU case volume relevant to pneumonia, this contextualizes whether 104 patients over ~5 years is expected or surprisingly low.

Can you justify the endpoint of your study? (e.g., change in local guidelines, COVID-19 impact, formulary change) why did you decide to study just these years?

7. Study period: There is need to address the COVID-19 overlap (2020–2022) must be addressed. Did you exclude COVID-19 pneumonia? Did the pandemic alter your ICU's antibiotic prescribing practices or microbiological documentation rates? These are major issues that needs to be addressed and that add to the context surrounding your study.

8. Lines 104 - 109: I think you need to make a good case for your exclusion criteria. How did you ensure you excluded all patients with Healthcare-associated pneumonia (HCAP)? did you exclude patients with nursing home residence, dialysis, or home IV therapy?

Also, immunocompromised patients i.e transplant recipients, HIV, chemotherapy e.t.c did you include or exclude? This affects pathogen spectrum dramatically. How did you deal with COVID-19 pneumonia? This is critical given your timeframe. And polymicrobial infections with non-targeted pathogens how were these handled? How did you handle incomplete microbiological or clinical records? These are critical areas you need to describe for your exclusion criteria.

9. Line 104: What clinical/radiological criteria distinguished CAAP from CAP? Its not clear how you distinguished these 2 from the data. Did you use a validated definition or an institutional one?Were CAP and CAAP managed under the same empirical antibiotic protocol or different ones? Justify why they are analyzed together or report whether you did a subgroup analysis separating them. These should be clearly stated in your methods section but are missing

10. Lines 140 - 142: For the sake of reproducibility, how did you define appropriateness? Appropriate = at least one prescribed antibiotic covers all documented pathogens based on in vitro susceptibility?Or based on guideline concordance?Or both? And who adjudicated appropriateness? clinician, pharmacist, independent reviewer? This information is crucial for your methods section.

11. Lines 145 - 154: Which goodness-of-fit measure was used? Hosmer-Lemeshow test?Nagelkerke R²? AUC/c-statistic?All of the above? Include this in the analysis section of the methods. Also, there is no mention of missing data here. Missing data handling is a major source of bias in retrospective studies and must be explicitly addressed.

12. Lines 148 - 149: Your multivariate model building is not completely described. Though the p<0.20 threshold for variable entry is stated, but several elements are missing:

- Maximum number of variables allowed in the model given sample size

- The standard rule of thumb is 1 variable per 10 events (EPV rule)

With n=104 and presumably modest AMC resistance rates, your event count may be very limited. This should be stated

- How did you handle collinear variables were handled at model entry

- Was backward, forward, or enter selection used after univariate screening?

- Were interaction terms considered?

please clarify all of these issues.

13. Lines 146 - 147: You correctly use non-parametric tests (Wilcoxon rank-sum) and report medians with IQR, this implies non-normal distribution. However, Was normality formally tested (Shapiro-Wilk) or assumed based on visual inspection (Q-Q plots, histograms)?It is important you state the basis for choosing non-parametric tests

14. Line 153: The R++ is not a conventional software used for this type of data analysis but is still acceptable. (i am not familiar with this software) May i know the justification for using this software (just curious)

15. Figure 2 has some issues namely:

A. Axes have no labels. Also, no Absolute Numbers (n) on the Bars or Axes. Percentages alone are misleading without denominators S. aureus showing 100% susceptibility to both agents looks identical to a finding based on 3 isolates vs. 30 isolates which the reader cannot tell. This is especially critical for the overall cohort bar where patients may have multiple pathogens. Add n= below each species name on the x-axis: e.g., Streptococcus pneumoniae (n=27) or include in the bar itself

B. No Error Bars or Confidence Intervals. For a primary endpoint in this study, point estimates without uncertainty measures are insufficient. The 81.5% AMC susceptibility for SP with no CI could represent a very wide range given small n. Either add 95% CI error bars to each segment, or present CIs in an accompanying table and reference it in the figure legend

C. Your abstract and methods use "3GC" (third-generation cephalosporins) while the figure uses "C3G" please be consistent.

D. The "Overall Cohort" Column Is Statistically Problematic as patients with co-infections (e.g., SP + HI) contribute isolates to multiple species columns AND to the overall cohort column. This means the overall cohort bar does not represent independent observations. It is unclear whether the overall bar represents:% of isolates that were susceptible (isolate-level analysis), % of patients whose empirical therapy was covered (patient-level analysis)

These are fundamentally different metrics and must be distinguished.

E. CAP vs. CAAP Breakdown Is Absent: Given that 57.7% of your cohort had CAAP and SA is likely overrepresented there, susceptibility profiles may differ meaningfully between CAP and CAAP. A single merged figure obscures this potentially important finding. You could split into two panels: Panel A = CAP | Panel B = CAAP or at minimum, acknowledge in the legend that CAP and CAAP are combine. Either remove the overall cohort bar and replace with a patient-level appropriateness figure, or add a clear footnote explaining the unit of analysis and how co-infections were handled.

16. Lines 256 - 356: The discussion appears to be merely descriptive rather than a critical synthesis that attempts to explain the heterogeneity across studies. So the discussion cites Sarr et al. (2025) and Hariri et al. (2017) but never critically reconciles the discrepancies between their findings and yours. Hariri et al. reported AMC susceptibility of 69.3% — substantially lower than your 86.5% and Sarr et al.'s 83.5%. This is a clinically meaningful difference of about 17 percentage points that could change empirical prescribing decisions. The discussion acknowledges the difference exists but does not explain it. Similarly, the independent risk factors for AMC resistance differ across all three studies:Your study: HI infection only, Sarr et al.: prior antibiotic exposure only, Hariri et al.: chronic respiratory disease, cancer, prior stroke, functional autonomy, prior antibiotic exposure. The discussion presents these as parallel findings without asking why they diverge. This is a significant analytical gap. Please build a critical synthesis that attempts to explain the heterogeneity across studies, acknowledges what remains unresolved, and explicitly states what this means for clinical practice and future research. The discussion should answer: "Given these inconsistencies across three single-center retrospective studies, what can clinicians actually conclude, and what study design would resolve the uncertainty?"

**********

6. PLOS authors have the option to publish the peer review history of their article (what does this mean?). If published, this will include your full peer review and any attached files.

If you choose “no”, your identity will remain anonymous but your review may still be made public.

**Do you want your identity to be public for this peer review?**  For information about this choice, including consent withdrawal, please see our Privacy Policy.

Reviewer #1: No

Reviewer #2: No

Reviewer #3: No

**********

[NOTE: If reviewer comments were submitted as an attachment file, they will be attached to this email and accessible via the submission site. Please log into your account, locate the manuscript record, and check for the action link "View Attachments". If this link does not appear, there are no attachment files.]

To ensure your figures meet our technical requirements, please review our figure guidelines: https://journals.plos.org/plosone/s/figures

You may also use PLOS’s free figure tool, NAAS, to help you prepare publication quality figures: https://journals.plos.org/plosone/s/figures#loc-tools-for-figure-preparation.

NAAS will assess whether your figures meet our technical requirements by comparing each figure against our figure specifications.

# Author comment: Author response to Decision Letter 1

**DOI:** 10.1371/journal.pone.0354339.r002

14 Jun 2026

RESPONSE TO REVIEWERS

To the Editor in Chief, to the Academic Editor, Mabel Kamweli Aworh, and to the reviewers.

We thank again all the reviewers and the editor for their helpful and insightful comments.

Please find enclosed the new revised version of our manuscript entitled: Third-generation cephalosporins are often unjustified in severe community-acquired pneumonia: insights from a retrospective French critical care cohort.

All the modifications suggested by the reviewers have been fully considered. Accordingly, a new version including all the requested modifications or explanations is provided (highlighted in yellow in the Revised Manuscript with Track Changes file). Additionally, a point-by-point response including a detailed rebuttal of all comments, criticisms, and revisions has been provided separately. These responses (R) are enclosed below and detail all the modifications provided in the new version of the manuscript.

We hope that this last modified version will be suitable for publication in the journal. As corresponding authors, we remain at your entire disposal if any further information is requested.

Best regards.

Quitterie Lostie de Kerhorr, MD

Jonathan Chelly, MD

Journal Requirements

When submitting your revision, we need you to address these additional requirements.

1. Please ensure that your manuscript meets PLOS ONE's style requirements, including those for file naming.

(R) We have carefully reviewed and applied PLOS ONE's style requirements throughout the manuscript, including file naming conventions, formatting of the main body, title page, authors, and affiliations.

2. In the online submission form you indicate that your data is not available for proprietary reasons and have provided a contact point for accessing this data. Please note that your current contact point is a co-author on this manuscript. According to our Data Policy, the contact point must not be an author on the manuscript and must be an institutional contact, ideally not an individual. Please revise your data statement to a non-author institutional point of contact, such as a data access or ethics committee, and send this to us via return email. Please also include contact information for the third-party organization, and please include the full citation of where the data can be found.

(R) We have updated our data availability statement to comply with the journal's policy. The new institutional contact point is the Délégation à la Recherche Clinique et à l'Innovation du Groupement Hospitalier Territorial du Var (DRCI GHT 83). They can be contacted for data access requests at support-recherche@drci-ght83.fr

3. Your ethics statement should only appear in the Methods section of your manuscript. If your ethics statement is written in any section besides the Methods, please move it to the Methods section and delete it from any other section. Please ensure that your ethics statement is included in your manuscript, as the ethics statement entered into the online submission form will not be published alongside your manuscript.

(R) The ethics statement has been exclusively moved to the "Study setting and population" subsection of the Methods section. It has been completely removed from all other sections of the manuscript.

4. Please include captions for your Supporting Information files at the end of your manuscript, and update any in-text citations to match accordingly. Please see our Supporting Information guidelines for more information: http://journals.plos.org/plosone/s/supporting-information.

(R) Captions for the Supporting Information files (S1 Table and S2 Table) have been appropriately added to the end of the manuscript, and all in-text citations have been updated to match this formatting.

5. If the reviewer comments include a recommendation to cite specific previously published works, please review and evaluate these publications to determine whether they are relevant and should be cited. There is no requirement to cite these works unless the editor has indicated otherwise.

(R) We have carefully evaluated all literature mentioned by the reviewers. Relevant publications have been added to our reference list and integrated into the Discussion to enrich the scientific context of our study.

Additional Editor Comments

In addition to addressing all reviewers' comments, please move the study limitations to the last paragraph of the Discussion section. In the conclusion section, please provide some recommendations based on the key study findings.

(R) We thank the Editor for these structural recommendations. The study limitations have been relocated to form the final paragraph of the Discussion section. Furthermore, we have expanded the Conclusion section to include specific recommendations for clinical practice and antimicrobial stewardship based on our key findings as follows:

“In this single-center retrospective cohort of ICU patients with severe CAP or CAAP due to S. pneumoniae, H. influenzae, and/or S. aureus, AMC showed high susceptibility rates and was frequently used appropriately, whereas 3GCs were often prescribed without microbiological justification. Given the inherent limitations of our study design, these findings cannot formally dictate an immediate shift in clinical guidelines. However, they highlight the need to question the systematic empirical use of broad-spectrum 3GCs, by conducting large-scale, randomized controlled trials to formally evaluate the clinical efficacy and safety of AMC as a first-line empirical alternative in severe CAP.”

Reviewer 1

1. The purpose of an abstract is to summarize the key findings in an easy way to understand please rephrase the following:

“AMC was prescribed empirically in 59% of cases and was appropriate in 86.9%”. – AMC was appropriate in 86.9% of cases, however it was only prescribed empirically in 59% of those cases.

“3GCs, prescribed in 20% of cases, were mostly unjustified (80%).”

(R) We thank the reviewer for pointing out this lack of clarity. We have rephrased these sentences in the Abstract to make the findings more straightforward. The text has been modified to: “AMC was the empirical treatment in 59% of cases, and this choice was microbiologically appropriate in 86.9% of these prescriptions. Conversely, 3GCs were prescribed empirically in 20% of cases, but this broad-spectrum choice was unjustified in 80% of those situations."

2. Under line 252:

Recommended beta-lactam class for CAP/CAAP

“(a) Appropriate use of AMC was defined as susceptibility to amoxicillin and/or AMC and/or oxacillin, depending on the identified pathogen. (b) Justified use of 3GC was defined as susceptibility to 3GC with resistance to amoxicillin and/or AMC and /or oxacillin, depending on the identified pathogen. Inappropriate therapy was defined as resistance to the evaluated empirical treatment. (c) Justified use of PTZ was defined as susceptibility to PTZ with resistance to amoxicillin and/or AMC and/or oxacillin and 3GC, depending on the identified pathogen”

I am having problems with these terminologies. Appropriate for AMC was defined, inappropriate for AMC was not (could mean the opposite of what appropriate could mean for AMC). Then B we changed it to justified- also defined, but no definition for unjustified, and inappropriate means resistance to the evaluated treatment- what evaluated treatment?

Same issue for C.

(R) We apologize for the confusion caused by the terminology. We have thoroughly revised these definitions in the Methods and the footnotes in Table 4 to ensure clarity and consistency.

Consequently, the text was modified in the Methods section as follows:

“Prescribed empirical therapy was classified as appropriate if the antibiotic effectively covered the isolated pathogen based on susceptibility testing, and inappropriate if the pathogen was resistant to the treatment. Among appropriate prescriptions, the use of a broader-spectrum agent (3GCs or PTZ) was classified as justified if the pathogen exhibited resistance to narrower-spectrum alternatives. Conversely, it was classified as unjustified when the empirical antibiotic was active, but a narrower-spectrum recommended option would have provided identical coverage, representing a situation of overtreatment.”

And the footnotes in Table 4 were modified to:

(a) Appropriate therapy: The empirical antibiotic prescribed covered the identified pathogen based on in vitro susceptibility.

(b) Inappropriate therapy: The identified pathogen was resistant in vitro to the empirical antibiotic prescribed.

(c) Unjustified therapy (overtreatment): The empirical antibiotic prescribed covered the pathogen, but a narrower-spectrum recommended alternative (e.g., AMC instead of a 3GC) would have been equally effective based on the susceptibility profile.

(d) Justified therapy: The use of a broader-spectrum agent (e.g., 3GC or PTZ) was necessary because the pathogen was resistant to narrower-spectrum alternatives (e.g., AMC).

Reviewer 2

The manuscript presents a retrospective study examining the unwarranted use of third-generation cephalosporins in patients with severe community-acquired pneumonia. This research significantly contributes to antibiotic stewardship, particularly in the context of the ongoing challenge of antibiotic resistance.

It is commendable that the authors adhered to STROBE guidelines in their methodology. While the study claims to have anonymized the collected data, the manuscript lacks detail on the deidentification process (which should be addressed), given that patient information in clinical settings is often not fully deidentified.

The operationalization of variables is well presented, alongside an account of the statistical methods employed and the necessary ethical approval. Results are presented clearly, and the discussion is robust. The study's limitations are addressed with proposed further research. The references cited are relevant and well-documented, further strengthening the manuscript's credibility. Overall, the paper is written in a clear and accessible manner. The authors have communicated the unjustified use of third-generation cephalosporins in the treatment of severe community-acquired pneumonia, emphasizing the need for scrutinizing antibiotic prescribing practices.

Upon clarification of how the collected data were anonymized, this research will be suitable for publication.

(R) We thank the reviewer for this important methodological comment. We have added a detailed description of the de-identification process in the "Data collection and outcome variables" section. Specifically, we clarified that patient data was extracted from electronic medical records and pseudonymized assigning a unique study identification number. The correspondence key linking patient identities to their study IDs was kept on a secured, hospital-hosted server accessible only to the primary investigators. All subsequent analyses were performed strictly on the de-identified dataset.

Reviewer 3

I want to commend the authors for their manuscript which in the current context of AMR is clinically relevant and timely. Antibiotic stewardship in severe CAP is a genuine priority in critical care medicine, and challenging entrenched prescribing habits is valuable. I have a few comments and suggestions which could help improve the context of the manuscript and increase value for the readers.

1. Title:

I think the title may be a bit sensational. Severe CAP (typically ICU-level) is a specific and high-acuity population where empiric broad-spectrum coverage is often precisely the standard of care due to diagnostic uncertainty on admission. The claim that third-generation cephalosporins (3GCs) are unjustified in this population is counterintuitive and risks being misleading without immediate clarification. This makes the title sensational in a way that could undermine credibility. Suggested "Third-generation cephalosporin use is frequently non-guideline-concordant in severe community-acquired pneumonia: findings from a French critical care cohort"

(R) We fully agree with the reviewer that the original title could be perceived as overly definitive, especially given the necessary broad-spectrum coverage initially required in ICU settings. Consequently, we have modified the title, as suggested by the reviewer.

2. Abstract (Lines 45 – 47):

The authors should be mindful that this is a Single-center, retrospective design and this language needs to be stressed in the conclusion. The conclusion as it reads is kind of broadly generalizable. A single-center study of 104 patients over about 5 years in one ICU carries significant selection bias and limits external validity. This must be flagged in the conclusion, not just buried in the methodology. I suggest that you conclude by saying "In this single-center retrospective cohort, AMC appeared to be a relevant empirical option... These findings require prospective multicenter validation before practice change can be recommended."

(R) We thank the reviewer for his pertinent remark. We have amended the conclusion in the Abstract as suggested by the reviewer, to explicitly highlight the single-center, retrospective nature of the study, thereby avoiding overstating the external validity of our findings.

Moreover, the Conclusion section of the main manuscript was also modified in the same way, as follows:

“In this single-center retrospective cohort of ICU patients with severe CAP or CAAP due to S. pneumoniae, H. influenzae, and/or S. aureus, AMC showed high susceptibility rates and was frequently used appropriately, whereas 3GCs were often prescribed without microbiological justification. Given the inherent limitations of our study design, these findings cannot formally dictate an immediate shift in clinical guidelines. However, they highlight the need to question the systematic empirical use of broad-spectrum 3GCs, by conducting large-scale, randomized controlled trials to formally evaluate the clinical efficacy and safety of AMC as a first-line empirical alternative in severe pneumonia.”

3. Abstract (Lines 30 – 33):

Your abstract does not mention the AMC resistance risk factor objective mentioned in your main manuscript. This is a significant inconsistency between your abstract and manuscript body that must be corrected.

(R) We thank the reviewer for raising this point. While we acknowledge that evaluating risk factors for AMC resistance was a secondary objective of our study, we deliberately chose not to include this specific analysis in the abstract for two main reasons. First, the strict 300-word limit imposed required us to prioritize our primary descriptive findings regarding overall susceptibility rates and the appropriateness of empirical prescriptions. Second, and most importantly, as we explicitly acknowledge in the manuscript, the limited sample size (specifically the low number of AMC-resistant events; n=14) considerably restricts the statistical power and robustness of our multivariable model. We felt that featuring this exploratory finding in the abstract might overstate its validity and potentially mislead the reader by presenting it as a definitive conclusion. Therefore, we prefer to detail this finding exclusively within the Results and Discussion sections of the main text, where its inherent limitations can be properly and fully contextualized. We hope the reviewer will understand and agree with this editorial and methodological choice.

4. Abstract (Lines 38 – 43):

Though the sample size is small, but confidence intervals are absent. Having 104 patients over nearly 5 years is a modest cohort. Susceptibility rates reported as point estimates (e.g., 81.5% for SP) with no confidence intervals are insufficient. Please add CIs to all primary endpoint results.

(R) We thank the reviewer for this pertinent statistical reminder. We completely agree that reporting point estimates of proportions without uncertainty measures is insufficient, particularly given our sample size. We have now calculated and added 95% Confidence Intervals (95% CIs) to all primary susceptibility outcomes (proportions) in the Abstract, the Results section, and the relevant figures and tables. For instance, the AMC susceptibility rate for

Attachment Submitted filename: Response to Reviewers.docx

# Peer review: Decision Letter 1

**DOI:** 10.1371/journal.pone.0354339.r003

**Authors:** Mabel Aworh

7 Jul 2026

Third-generation cephalosporin use is frequently non-guideline-concordant in severe community-acquired pneumonia: findings from a French critical care cohort.

PONE-D-26-15039R1

Dear Dr. Chelly,

We’re pleased to inform you that your manuscript has been judged scientifically suitable for publication and will be formally accepted for publication once it meets all outstanding technical requirements.

Within one week, you’ll receive an e-mail detailing the required amendments. When these have been addressed, you’ll receive a formal acceptance letter and your manuscript will be scheduled for publication.

An invoice will be generated when your article is formally accepted. Please note, if your institution has a publishing partnership with PLOS and your article meets the relevant criteria, all or part of your publication costs will be covered. Please make sure your user information is up-to-date by logging into Editorial Manager at Editorial Manager® and clicking the ‘Update My Information' link at the top of the page. For questions related to billing, please contact billing support.

If your institution or institutions have a press office, please notify them about your upcoming paper to help maximize its impact. If they’ll be preparing press materials, please inform our press team as soon as possible -- no later than 48 hours after receiving the formal acceptance. Your manuscript will remain under strict press embargo until 2 pm Eastern Time on the date of publication. For more information, please contact onepress@plos.org.

Kind regards,

Mabel Kamweli Aworh, DVM, MPH, PhD. FCVSN

Academic Editor

PLOS One

Additional Editor Comments (optional):

Reviewers' comments:

Reviewer's Responses to Questions

**Comments to the Author**

1. If the authors have adequately addressed your comments raised in a previous round of review and you feel that this manuscript is now acceptable for publication, you may indicate that here to bypass the “Comments to the Author” section, enter your conflict of interest statement in the “Confidential to Editor” section, and submit your "Accept" recommendation.

Reviewer #2: All comments have been addressed

Reviewer #3: All comments have been addressed

**********

2. Is the manuscript technically sound, and do the data support the conclusions?

The manuscript must describe a technically sound piece of scientific research with data that supports the conclusions. Experiments must have been conducted rigorously, with appropriate controls, replication, and sample sizes. The conclusions must be drawn appropriately based on the data presented.

Reviewer #2: Yes

Reviewer #3: Yes

**********

3. Has the statistical analysis been performed appropriately and rigorously?

Reviewer #2: Yes

Reviewer #3: Yes

**********

4. Have the authors made all data underlying the findings in their manuscript fully available?

The PLOS Data policy requires authors to make all data underlying the findings described in their manuscript fully available without restriction, with rare exception (please refer to the Data Availability Statement in the manuscript PDF file). The data should be provided as part of the manuscript or its supporting information, or deposited to a public repository. For example, in addition to summary statistics, the data points behind means, medians and variance measures should be available. If there are restrictions on publicly sharing data—e.g. participant privacy or use of data from a third party—those must be specified.

Reviewer #2: Yes

Reviewer #3: Yes

**********

5. Is the manuscript presented in an intelligible fashion and written in standard English?

PLOS ONE does not copyedit accepted manuscripts, so the language in submitted articles must be clear, correct, and unambiguous. Any typographical or grammatical errors should be corrected at revision, so please note any specific errors here.

Reviewer #2: Yes

Reviewer #3: Yes

**********

6. Review Comments to the Author

Please use the space provided to explain your answers to the questions above. You may also include additional comments for the author, including concerns about dual publication, research ethics, or publication ethics. (Please upload your review as an attachment if it exceeds 20,000 characters)

Reviewer #2: Thank you to the authors for submitting a revised version of the manuscript. It is evident in this revised version that the authors have made considerable effort to address the issues raised in the initial review.

Overall, the authors have implemented the recommendations provided, significantly strengthening the scientific rigor of the manuscript.

Reviewer #3: Again i want to commend the authors for the rigorous work they did with this research and the manuscript. Authors have satisfactorily responded to all my comments. I have no futher comments or questions.

**********

7. PLOS authors have the option to publish the peer review history of their article (what does this mean?). If published, this will include your full peer review and any attached files.

If you choose “no”, your identity will remain anonymous but your review may still be made public.

**Do you want your identity to be public for this peer review?**  For information about this choice, including consent withdrawal, please see our Privacy Policy.

Reviewer #2: No

Reviewer #3: No

**********

# Editor report: Acceptance letter

**DOI:** 10.1371/journal.pone.0354339.r004

**Authors:** Mabel Aworh

PONE-D-26-15039R1

PLOS One

Dear Dr. Chelly,

I'm pleased to inform you that your manuscript has been deemed suitable for publication in PLOS One. Congratulations! Your manuscript is now being handed over to our production team.

At this stage, our production department will prepare your paper for publication. This includes ensuring the following:

* All references, tables, and figures are properly cited

* All relevant supporting information is included in the manuscript submission,

* There are no issues that prevent the paper from being properly typeset

You will receive further instructions from the production team, including instructions on how to review your proof when it is ready. Please keep in mind that we are working through a large volume of accepted articles, so please give us a few days to review your paper and let you know the next and final steps.

Lastly, if your institution or institutions have a press office, please let them know about your upcoming paper now to help maximize its impact. If they'll be preparing press materials, please inform our press team within the next 48 hours. Your manuscript will remain under strict press embargo until 2 pm Eastern Time on the date of publication. For more information, please contact onepress@plos.org.

You will receive an invoice from PLOS for your publication fee after your manuscript has reached the completed accept phase. If you receive an email requesting payment before acceptance or for any other service, this may be a phishing scheme. Learn how to identify phishing emails and protect your accounts at https://explore.plos.org/phishing.

If we can help with anything else, please email us at customercare@plos.org.

Thank you for submitting your work to PLOS ONE and supporting open access.

Kind regards,

PLOS ONE Editorial Office Staff

on behalf of

Dr. Mabel Kamweli Aworh

Academic Editor

PLOS One
